# A strategy for sequence control in vinyl polymers via iterative controlled radical cyclization

**DOI:** 10.1038/ncomms11064

**Published:** 2016-03-21

**Authors:** Yusuke Hibi, Makoto Ouchi, Mitsuo Sawamoto

**Affiliations:** 1 Department of Polymer Chemistry, Graduate School of Engineering, Kyoto University, Katsura, Nishikyo-ku, Kyoto 615-8510, Japan; 2 Precursory Research for Embryonic Science and Technology (PRESTO), Japan Science and Technology Agency (JST), 4-1-8 Kawaguchi, Saitama 332-0012, Japan

## Abstract

There is a growing interest in sequence-controlled polymers toward advanced functional materials. However, control of side-chain order for vinyl polymers has been lacking feasibility in the field of polymer synthesis because of the inherent feature of chain-growth propagation. Here we show a general and versatile strategy to control sequence in vinyl polymers through iterative radical cyclization with orthogonally cleavable and renewable bonds. The proposed methodology employs a repetitive and iterative intramolecular cyclization via a radical intermediate in a one-time template with a radical-generating site at one end and an alkene end at the other, each of which is connected to a linker via independently cleavable and renewable bonds. The unique design specifically allowed control of radical addition reaction although inherent chain-growth intermediate (radical species) was used, as well as the iterative cycle and functionalization for resultant side chains, to lead to sequence-controlled vinyl polymers (or oligomers).

## Abstract (web summary)

The ultimate control in polymer synthesis, precise monomer sequencing, is a highly sought after but complex problem. Here, the authors employ a repetitive and iterative intramolecular cyclization reaction, via a radical intermediate, to generate sequence-controlled vinyl oligomers.

## Graphical abstract

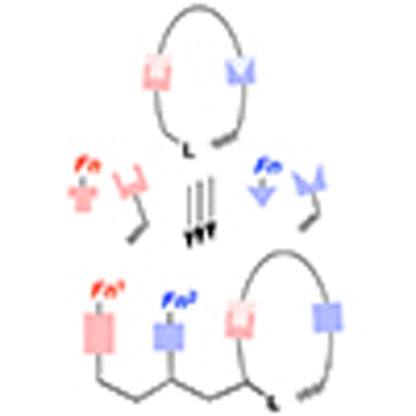

In living cells, the genetic information in DNA is transcribed to RNA which is then translated to produce a peptide chain of defined sequence^1^. In these transcription–translation processes, elaborate template mechanisms are critical, particularly in the translation from mRNA to peptides. Thus, a parent mRNA conjugates with a ribosome as a reaction field, which precisely controls the propagation reaction, that is, the amidation of a growing carboxylate terminus with the amine in a particular amino acid monomer. Namely, a terminal unit and a monomer (an amino acid) are introduced on mRNA via a particular transfer RNA (tRNA) that recognizes and then associates with a three-letter ‘codon', or a triad sequence of nucleic acid residues in the mRNA via complementary hydrogen bonds (Fig. 1). It is notable that only a single pair of a terminus unit and a monomer are introduced into this reaction field to control a one-time selective and specific amidation step (‘single amidation' or the iterative propagation of one monomer unit): other amino acids (monomers) are apathetic to this particular propagation step until they are recognized next to the terminal. The order of amino acids to be introduced into ribosome is programmed on the basis of the codons in mRNA originating from the genetic information stored in a mother DNA. After the single amidation, the tRNA clinging to a pre-terminus unit of the growing protein is removed from the ribosome, which moves forward to the next codon along the mRNA backbone to make a new space for the tRNA to recognize the next amino acid. This cycle thus consists of three consecutive stepwise reactions, ‘single amidation' (single-step propagation), ‘removal of tRNA' (cleavage of template), and ‘recognition of amino acid' (introduction of a next monomer), by which the sequence of protein is perfectly controlled, even though as many as 20 amino acids are employed as ‘comonomers' with near identical reactivities. The controlled sequence of amino acids thereby allows the formation of well-defined structures of proteins leading to their effective and smart functions, and the ‘sequence-fidelity' to the functions is most glaringly apparent in that only slight difference in sequence could provide totally different characters of proteins. Needless to say, therefore, sequence is indubitably an essential structural factor for natural polymers. In synthetic macromolecules such as polymethacrylates, the sequence herein implies the order of pendent functionality along a carbon-based (not a polyamide-based) polymer backbone.

For synthetic polymers, living polymerization techniques have allowed control of initiation and propagation without any irreversible termination and chain transfer reactions, leading to the precision synthesis of well-defined polymer architectures as well as control of molecular weight, molecular weight distribution, and terminal groups^2^. Especially, living radical polymerization has now been widely employed in various fields, even beyond polymer science, because of the superior, user-friendly usability and versatility, and the combination of abundant functionalized monomers has allowed the precision synthesis of tailor-made functional polymers^3^. We can now design well-defined functional polymers directed towards structure-oriented functions.

These synthetic polymers by chain-growth polymerization, however precisely synthesized, are still no match for biopolymers in structural elaborateness and functions, entirely because their repeat-unit sequence cannot be regulated as precisely as with their biological counterparts. The growing species in a chain-growth polymerization under usual conditions can, by definition, instantly and continuously reacts with monomers, rendering an iterative single-monomer propagation (propagation one by one) practically very difficult though not impossible. In this regard, as demonstrated by the *in vivo* peptide synthesis as well as by *in vitro* syntheses on resin^4^ or in solution^5^, using monomers applicable in step-growth polymerization might be best suited for sequence regulation where a stepwise bond-formation reaction can be independently and sequentially repeated. Indeed, as far non-vinyl polymer type of sequence-controlled macromolecules, some approaches with iterative coupling along with deprotection of reactive cite have been reported, and sequence control for high molecular weight polymer has been realized^6^,^7^,^8^,^9^,^10^,^11^.

Given this recognition, intense efforts have been directed towards sequence control in synthetic polymers^12^,^13^,^14^, particularly those by radical and other chain-growth polymerizations^15^,^16^,^17^,^18^, but sequence-control methodologies are still limited, only allowing position control^19^ and periodic or repeated patterns 17,18,20,21,22, with little examples of sequence-originating functions^23^,^24^,^25^,^26^,^27^,^28^,^29^. The now classical ‘template polymerizations' seem to have provided limited success^30^. Efforts to control the reactivity with designed template are known^16^,^31^,^32^, but control of the reactivity ratio is generally impracticable to approach sequence control for longer chain.

In the contemporary living radical polymerizations, the reversible activation of a dormant species into an active growing end allows the controlled radical propagation without irreversible side reactions. In regard to the sequence control by iterative single-monomer propagation as discussed above, it is important to note that the involvement of dormant species would suppress severe propagation of active species in chain-growth mechanism. However, even by using living radical polymerization, repetition of the single-monomer addition via an apparently stepwise-activated dormant species is definitely impossible. For example, the propagation step in metal-catalysed living radical polymerization (or atom-transfer radical polymerization; ATRP)^33^,^34^ is basically identical to the carbon–carbon bond formation by the Kharasch addition^35^; but even with an equimolar mixture of a dormant end and a monomer, frequent oligomerizaion is very difficult to suppress. Single-monomer addition could be realized under specific conditions, such as the use of an excess amount of halogen-based initiator or of a non-conjugated monomer to give a less active dormant end, but these conditions are less suited for repeating the process.

To approach the iterative single-monomer addition with vinyl monomers, Huang and co-workers focused attention on ally alcohol [CH_2_=CH(CH_2_OH)] as a key monomer for the ATRP process^36^. The non-conjugated monomer gives an inactive carbon–halogen bond via the radical addition to give a single-monomer adduct [–CH_2_–CH(*CH*_*2*_*OH*)–Br], and the oxidation of the side chain followed by esterification allows the transformation of the pendent alcohol into an acrylate [–CH_2_–CH(*COOR*)–Br] that, as a conjugated ester, can now be activated into a growth-active radical upon redox ATRP catalysis. Thus, the iterative cycle of ‘single (non-conjugated) monomer addition', ‘pendent oxidation' and ‘esterification' would possibly give sequence-regulated acrylic polymers. However, the efficiency in the allylic addition is not quantitative high (<80 %), and the iterative three-step cycle has yet been achieved so far.

In this paper, aiming at sequence-controlled vinyl polymers and oligomers, we propose a new strategy to approach repetition of the single-monomer addition via a carbon-centred radical species on the basis of our metal-catalysed living radical polymerization. Crucial for this approach are as follows: how to control single-monomer addition; how to repeat the addition process; and how to incorporate side chain functionalities.

## Results

### Synthetic approach

Toward control of the single-monomer addition, we propose a templated-cyclization between a carbon–halogen bond (a dormant radical source) and a conjugated vinyl monomer both attached to the terminals of a cyclic spacer (template) architecture. With this construction, the radical source is activated under metal catalysis to generate a carbon radical that is to intramolecularly react with the conjugated alkene moiety to complete a selective and efficient cyclization. The radical addition, in turn would regenerate a potentially active carbon–halogen bond due to the conjugated side chain of the alkene moiety.

A carefully selected metal catalyst should ensure the selective radical formation and the subsequent cyclization, but the efficient catalysis requires diluted reaction conditions, to suppress further chain growth as well as irreversible unfavourable bimolecular radical reactions (for example, disproportionation and coupling). Equally important and unique to this approach, we designed a new mechanism in which the template embedded in the resultant cyclic structure is forward transferred, so as to realize the iteration of the addition reaction as inspired by the protein expression in nature where ribosome migrates to repeat amidation (see Fig. 2a). For the migration, the obtained endocyclic structure (closed ring) is ‘partially' cleaved afterward to generate an acyclic form (cleaved ring), thus a new vinyl group being introduced to reconstruct the chance of next cyclization reaction from opened ring. Herein S_N_2-type reaction is favoured for the cleavage, since it would allow modification of the side chain with functionality, while the linker is cleaved. For these criteria, two special cleavable linkages were selected for the linker, namely, selectively ‘cleavable' and ‘renewable' bonds under orthogonal conditions. Such bonds would allow migration of situation for templated radical addition, hopefully like bipedal walking^37^.

### Design of two types of cleavable and renewable bonds

**Figure 1:**
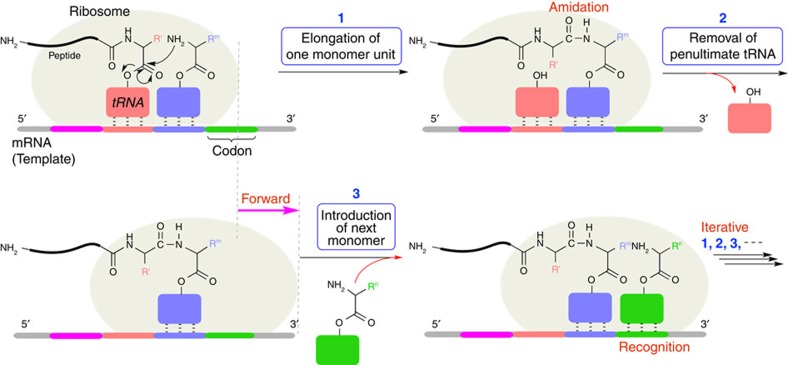
Schematic illustration of growing peptide chain in ribosome. The order of amino acid is programmed by codon in mRNA through the carboxylate-tRNA. The amidation takes place between the terminal carboxylate-tRNA and the amine group in the next amino acid monomer along with removal of the penultimate tRNA. The iterative cycle allows sequence-controlled propagation.

To approach this strategy, an inimer (initiator-monomer) was first designed: an initiator in metal-catalyzed living radical polymerization (carbon–halogen bond) and a methacrylate unit were introduced at both terminals in one molecule. Two ‘cleavable but renewable bonds', orthogonal to each other, were embedded between the two moieties as the special linker. To realize the expected process shown in Fig. 1, the following features are required for the two bonds: one bond should be selectively cleaved without damaging another as well as carbon–halogen bond (selective cleavage); they should be cleaved under attack of reactant like S_N_2 reaction for side-chain functionalization (S_N_2-type cleavage); the bonds should be quantitatively regenerated from the section after the cleavage through reaction with some molecule carrying vinyl group, therefore vinyl group can be dangled through the bond (quantitative regeneration); and they should survive during metal-catalyzed radical addition reaction (robustness).

**Figure 2:**
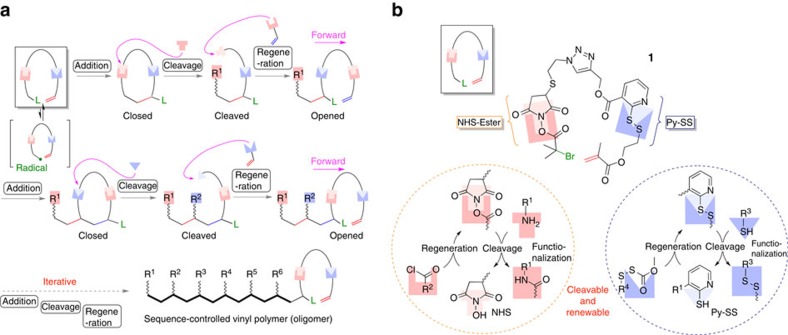
Conceptual scheme allowing iterative cyclization and the molecular structure of inimer 1 carrying two types of cleavable/renewable bonds. (**a**) Single-monomer addition can be controlled via cyclization to give ‘closed' structure. (**b**) To repeat the cyclization, one of cleavable/renewable bonds is cleaved (‘cleaved'), followed by regeneration of the bond carrying next vinyl group (‘opened'). NHS-Ester and Py-SS are embedded between the radical generator and conjugated vinyl group as the cleavable/renewable bonds in **1**.

Given these features, we selected *N*-hydroxysuccinimidyl ester (NHS-Ester)^38^ and *ortho*-pyridyl disulfide (Py-SS)^39^ as the ‘orthogonally' cleavable and renewable bonds (Fig. 2b). The bond of NHS-Ester can be cleaved upon the attack of a primary amine (R^1^–NH_2_) to give an amide and *N*-hydroxysuccinimide (NHS). From NHS, the NHS-Ester bond can be easily regenerated via esterification with an acid halide compound (for example, R_2_COCl). The bond of Py-SS can also be cut with an alkyl thiol (R^2^–SH) to give a disulfide and 2-mercaptopiridine (Py-SH), and the resulting thiol can react with an activated disulfide, R_4_SSCOOCH_3_, carrying an electron-withdrawing group adjacent to the S–S bond in the presence of triethylamine, to regenerate the Py-SS bond. As shown in Fig. 2b, therefore, the inimer (initiator and monomer) **1** was designed and constructed where a carbon–bromine (C–Br) bond (initiator or a radical precursor for living radical polymerization) and a methacrylate were connected through the two unique bonds, NHS Ester and PySS.

To realize the concept, quantitative cleavage and regeneration are required for the two cleavable but renewable bonds. Thus, the feasibility for the concept was first studied with the corresponding model compounds, that is, methacrylates carrying NHS-Ester (**NHS–MA**) and Py-SS (**PySS–MA**) in the side chains, which can be synthesized in the process of synthesis of **1** (see Supplementary Methods).

**Figure 3:**
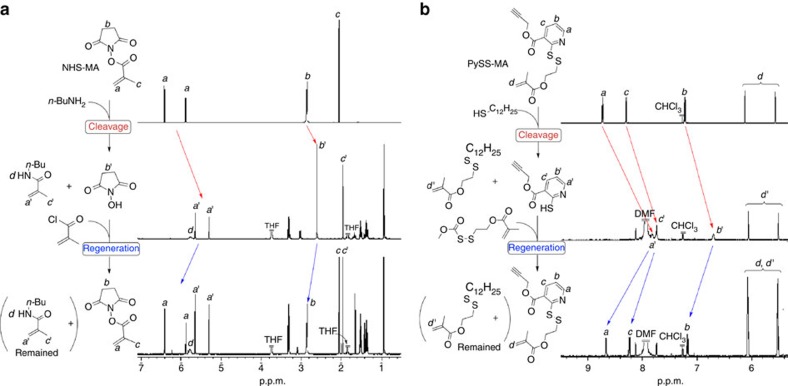
^1^H NMR-monitored model reactions for cleavage and regeneration of NHS-Ester and Py-SS. (**a**) Methacrylate-based compounds carrying NHS-Ester (NHS-MA) and (**b**) Py-SS (PySS-MA) were used as the model compound. Each model reaction was continuously performed without isolation of the cleaved product in one-pot synthesis way.

Figure 3 shows ^1^H NMR spectra for sequential reactions with model methacrylates (**NHS–MA** and **PySS–MA**) to demonstrate quantitative cleavage and regeneration of the two special bonds. Note that these spectra were obtained by *in situ* direct ^1^H NMR analysis on reaction mixtures, in one pot and without any isolation.

The NHS-Ester bond in **NHS–MA** was in fact cleanly cleaved with *n*-butyl amine (*n*-BuNH_2_) to give butyl methacrylamide and NHS, which was confirmed by quantitative peak shift of the vinyl protons (*a* → *a'*) and methylene protons in the NHS cyclic (*b* → *b'*) as well as the emergence of the amide peak (*d*). Subsequently, methacrylic chloride and Et_3_N were added into the solution, and the starting compound (**2**) and remaining unreacted butyl methacrylamide were observed, showing that the NHS bond in **NHS–MA** was first cleaved but was then quantitatively regenerated *in situ* just upon addition of an amine and an acid halide. Note that unreacted *n*-butyl amine remained even for the regeneration process since an excess of the amine was added. The residual amine could react with methacryloyl chloride to give methacrylamide, and indeed peak intensities from the amide protons (*a'* and *c'*) were enhanced.

The Py-SS bond in **PySS–MA** was also quantitatively cleaved into 2-mercaptopiridine (PySH) and a disulfide upon treatment with dodecanethiol (C_12_H_25_SH) and a catalytic amount of acetic acid, as supported by upfield shifts of the pyridine protons (*a*, *b*, and *c* → *a'*, *b'*, and *c'*, respectively). A methacrylate carrying an ‘activated' disulfide was then added *in situ* in the presence of Et_3_N. The pyridine protons (*a'*–*c'*) in **PySS–MA** disappeared, while Py-SS (peaks *a*–*c*) re-appeared, both quantitatively. The increased peak intensity of the dangling methacrylate (*d*) would further support the regeneration of Py-SS, though the peak overlap with **PySS–MA** (*d'*) hampered quantitative confirmation. In the model reaction, the residual dodecanethiol could react with the activated disulfide, nevertheless quantitative cleavage and regeneration for the Py-SS bond were confirmed in the model reactions.

Thus, the two bonds, NHS-Ester and Py-SS, are both suitable for orthogonally cleavable and renewable functions.

### Quantitative and selective cleavage of NHS-Ester and Py-SS

Following the separate model reactions for the orthogonal cleavage, another model reaction was examined with the inimer **1**, where the two cleavable bonds are placed within a single molecule. Obviously, the success of this reaction is essential to establish optimum reaction conditions for an orthogonal quantitative cleavage without damaging the other cleavable bond, as well as the carbon–halogen bond for radical generation, both within the same molecule. For example, the latter might react with the added amine or the added thiol for cleavages, and thus milder cleavage conditions would be required.

First, the cleavage of the NHS Ester bond was run with *n*-butyl amine (*n*-BuNH_2_). As it turned out, the use of 2-hydroxypyridine (2-HP) as the catalyst for the alkyl amine was effective, to allow the reaction proceeding even at 0 °C. Comparison of the ^1^H NMR spectra shows that, upon addition the base mixture, the methyl protons in **1** adjacent to NHS-ester clearly shifted (*a* → *a'*) and that new peaks (*o* and *p*) appeared, most likely indicative of an amide (Supplementary Fig. 1). Other peaks remained unchanged both in position and in intensity throughout the reaction.

For the cleavage of the Py-SS bond with a thiol, acetic acid (AcOH) as a catalyst was effective. Upon addition of 1-butanethiol (*n*-BuSH) into a solution of **1** with trace AcOH, selective and quantitative cleavage apparently proceeded: the pyridine protons, closer to the cleavage site, clearly shifted downfield (*h*, *i* and *j* → *h''*, *i''* and *j''*, respectively), whereas the methyl protons (*a''*) hardly shifted.

### Intramolecular radical addition with inimer 1

**Figure 4:**
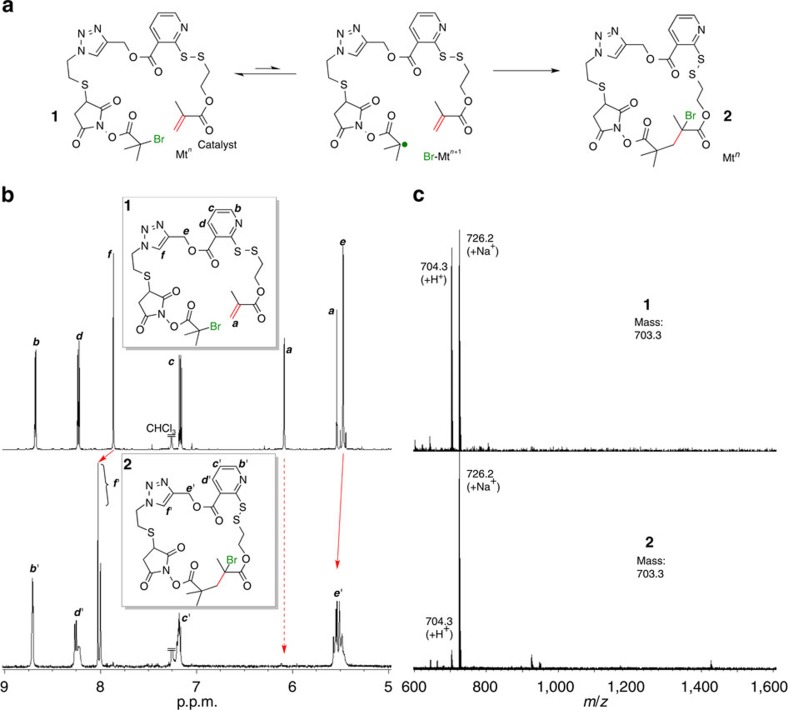
Single-monomer addition with 1 via radical cyclization. The progress of cyclization (**a**) was characterized with ^1^H NMR (**b**) and MALDI–TOF–MS (**c**) spectrum in comparison of the product (**2**) with **1** without any purification. The reaction condition is as follows: [**1**]/[CuBr_2_]/[MeO-bpy]=2.5/0.18/0.36 mM in the presence of Cu(0) in cyclohexanone/toluene (2/3 v/v) at 60 °C for 20 h.

In the proposed process (Fig. 2), the first step to be controlled is the metal-catalyzed intramolecular radical cyclization within **1** (Fig. 4a). In contrast to common cyclizations on the basis of Kharasch addition, where a transformation from active alkyl halide to inactive one is essential to progress the reaction^40^, the intramolecular reaction within **1** was designed to give active carbon–bromine bond in the product (**2**) for subsequent repetition of the radical reaction. Thus, the control is more challenging, and highly efficient catalysis even under diluted condition would be required. Besides, the reaction needs to be precisely controlled without scission of the two cleavable bonds as well as irreversible bimolecular radical terminations, which would also cause difficulty in selection of the condition. With these in mind, we screened the radical addition conditions in terms of catalyst, solvent, and reagent concentrations (Supplementary Table 1). A successful intramolecular addition calls for the complete consumption of the vinyl moiety (by ^1^H NMR), without altering the molecular weight of the substrate before and after the reaction (by matrix-assisted laser desorption/ionization–time of flight mass spectrometry (MALDI–TOF–MS)). In contrast, complicated NMR and MALDI–TOF–MS spectra would emerge, if unfavourable reactions occur, such as in-advance cleavage of the linking bonds, bimolecular radical termination (disproportionation and/or coupling) and oligomerization of the alkene units.

First runs employed ruthenium complexes such as Ru(Cp*)Cl(PPh_3_)_2_ and Ru(Ind)Cl(PPh_3_)_2_, active catalysts for radical addition^41^ and living radical polymerization^42^,^43^ in toluene at 60 °C under diluted conditions ([**1**]_0_=10 mM). Unfortunately, both catalysts cleaved the PySS linker in parallel with radical addition, probably by coordination onto the pyridine rather than radical generation (Entry 1 and 2 in Supplementary Table 1) (The peak of alkene protons in 1 split upon the treatment, while the integrated peak area unchanged).

Copper catalysts were then employed with some success. For example, 2,2′-bipyridyl (bpy) was used in conjunction with CuBr and CuBr_2_ in anisole (Entry 3). The cycloaddition apparently occurred, as indicated by a disappearance of the vinyl protons with no mass change of the substrate and the product. No S–S bond cleavage was detected in MALDI–TOF–MS spectrum, most likely thanks to the tighter chelate coordination of the bpy ligand favourable to copper rather than the PySS site^44^. However, the conversion was as low as 39%.

To improve catalyst activity, other solvents were also examined. Aprotic polar solvents such as DMF and DMSO gave higher conversions, but the MS peaks were complicated and different from that assumed for the expected adduct (Entries 4 and 5). On the other hand, a higher conversion (57%) into the cycloadduct was achieved with cyclohexanone (Cy) solvent (Entry 6). Minor MALDI–TOF–MS signals were also observed, however, indicative of HBr elimination from **1** as well as oligomerization.

Eventually, a near quantitative yield (*ca.* 100%; Entries 9 and 10) was achieved with 4,4′-dimethoxy-2,2'-bipyridyl (MeO-bpy)^45^ coupled with CuBr_2_ and Cu(O) (wire)^46^ in Cy/toluene (2/3 v/v) mixed solvent, without damaging the PySS site. Conversion was slightly lower (92%) in Cy solvent (Entry 8), and much lower (73 %) with MeO-bpy/CuBr_2_ but without Cu(O) (Entry 7). Thus, under the best conditions, the vinyl group completely disappeared (Fig. 4b; ^1^H NMR), and two peaks of 704.3 and 726.2 *m/z* were detected MALDI–TOF–MS, corresponding to the H^+^ and the Na^+^ adducts of **2**, respectively (Fig. 4c). The reaction mixture was passed through a pad of silica gel to remove catalysts, providing quantitative formation of the closed ring **2**. Interestingly, peaks from triazole protons (*f*) clearly shifted to lower magnetic field after the addition reaction, though it is far from the reaction site, which was likely due to change in the environment and ring-strain on the ring formation.

### Selective cleavage of one linker in first closed ring

The cleavage of the NHS-ester linker was done for the closed ring **2** with *n*-butyl amine in the presence of 2-HP in CH_2_Cl_2_ at 0 °C. Contrary to our expectation from the results of the model reaction (see Fig. 3), the mass spectrum (Supplementary Fig. 2) of the resultant solution did not indicate formation of the amine adduct through cleavage of the NHS-ester linker. From value of the mass peak, the product is probably the HBr-eliminated from the amine adduct intermediate. The unfavourable reaction would be specific to the ring structure, because such halogen elimination did not occur in the model reaction, as discussed above. In the structure of **2**, the carbon adjacent to the bromine (C–Br) is located close to the carbonyl group of the NHS-ester via two carbons, and thus the zwitter-ion intermediate may undergo the bromine substitution through transitional 5-membered ring state, rather than NHS-ester cleavage giving the NHS group. We speculated some routes for the unexpected reaction (Supplementary Figure 3), but abandoned further study of the mechanism.

**Figure 5:**
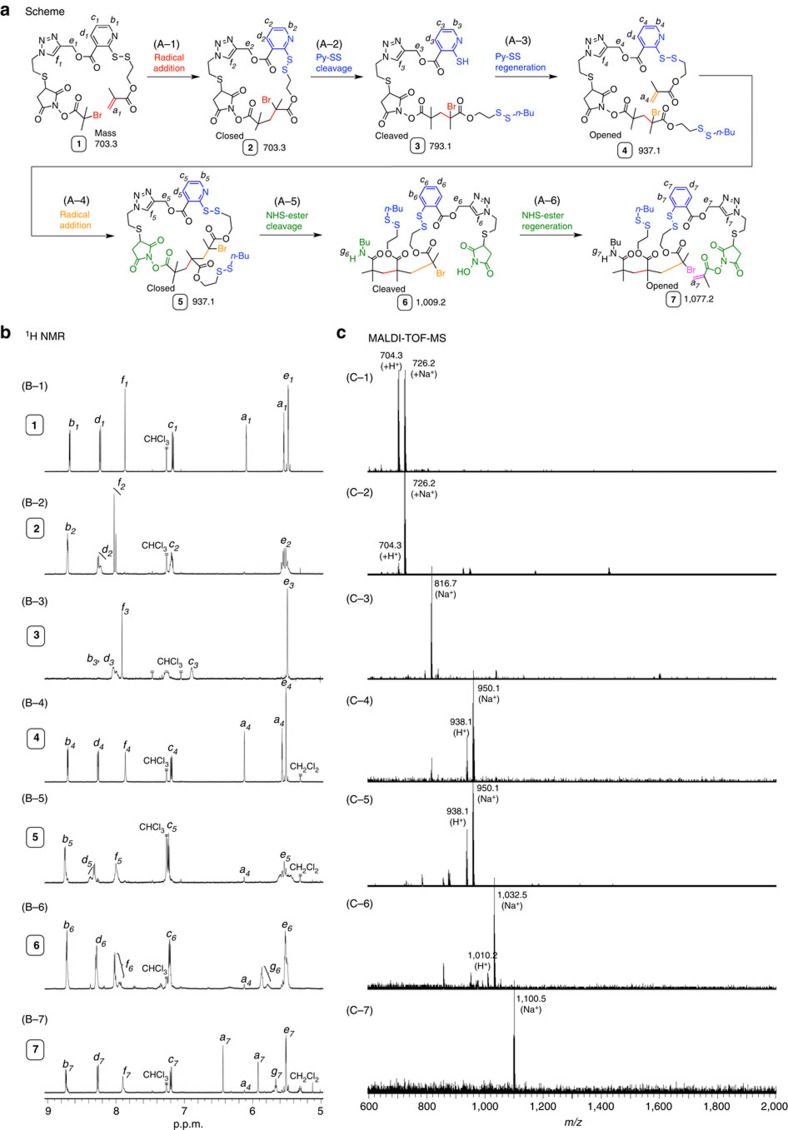
Two cycles of radical addition, cleavage, and regeneration. The overall scheme is shown in (**a**) the first cycle is for cleavage and regeneration of PySS and the second is for NHS-Ester. The progress of cyclization was characterized by ^1^H NMR (**b**) and MALDI–TOF–MS (**c**). See Supplementary Methods for the detailed conditions.

To settle the issue that the halogen substitution unfortunately occurs in the amine addition for **2**, the cleavage order was changed for the SS-pyridine site to be cleaved before the NHS-ester: thereby the NHS-ester site would be distanced from the carbon–halogen bond. Therefore, the SS pyridine in **2** (first Closed Ring) is first cleaved to prepare ‘first Cleaved Ring' [(A-2) in Fig. 5], followed by regeneration of the cleavable bond attaching vinyl group to construct ‘first Opened Ring' (A-3). After the radical addition with the opened ring compound (A-4), the NHS ester in the resultant ‘second Closed Ring' would be farer from the carbon–halogen bond (with four carbons) than in **2** (with two carbons). It was expected that the closed ring compound would be safely transformed into ‘second Cleaved Ring' through NHS-ester cleavage without the bromide substitution, successfully to lead to the opened form (second Opened Ring).

Thus, *n*-butanethiol (C_4_H_9_SH) was added into the solution of closed ring **2** in the presence of catalytic amount of acetic acid to cleave the SS-pyridine bond [(A-2) in Fig. 5]. The resultant solution was only evaporated and the residue was directly measured with ^1^H NMR. As the result, Py-SS was selectively and quantitatively cleaved to give cleaved ring **3**, which was confirmed by clear peak shift for protons of pyridine (*b*_*3*_–*d*_*3*_), methylene spacer (*e*_*3*_) and triazole (*f*_*3*_). Subsequently, 2-(methoxycarbonyldisulfanyl)ethyl methacrylate was injected for **3** to re-introduce the SS-pyridine linker carrying methacrylate group (A-3), and the reaction mixture was then passed through a pad of neutral silica gel to remove unnecessary reagents/catalysts. As expected, opened ring **4** was certainly generated: the peaks of methacrylate vinyl protons (*a*_*4*_) appeared at reasonable integration ratios as well as those of protons (*b*_*4*_-*f*_*4*_: B-4). The generation of **3** and **4** was also supported by MALDI–TOF–MS (C-3 and C-4), and the weight yield of pure opened ring **4** was 92%, based on starting material **1**.

### Second single-monomer addition

The opened ring **4** was applied for the next (second) ring-closing reaction via radical addition to be converted into the closed ring **5**. Unfortunately, same condition as for the first ring-closing reaction from **1** (opened) to **2** (closed) resulted in limited conversion of the vinyl group (∼80% by ^1^H NMR), and the product show extra peaks from disproportionation in addition to the ideal ones in MALDI–TOF–MS spectrum. This is likely due to that reactivity of carbon–bromine bond in **4** is different from that in the previous opened ring (**1**) likely due to difference in structure strain between the two ring structures. Thus, condition was tuned to suppress the side reactions by changing catalyst amount, temperature, and solvent. Consequently, decreasing amount of the catalyst and lower temperature were effective to accomplish almost quantitative consumption of vinyl group, and the conversion of the vinyl group reached 95% (by ^1^H NMR, only 5% vinyl group was remained) in 20 h (B-5 in Fig. 5). In MALDI–TOF–MS spectrum, peaks from the ideal product **5** were observed for 938 *m/z* (+H^+^) and 960 *m/z* (+Na^+^) without any other distinct peaks (C-5).

### Second selective cleavage and regeneration

As described above, when amine was added for the closed ring **2**, undesirable bromine substitution was incurred rather than the aimed cleavage of the NHS-ester due to the close spacer (with two carbons) between the carbonyl carbon in the NHS-ester and the carbon adjacent to bromine. The spacer in the closed ring **5** is longer (with four carbons) than in **2** (with two carbons), and thus it was expected that cleave of the NHS-ester successfully proceeded similar to the model reaction. In fact, after addition of *n*-butyl amine for the solution of **5** in the presence of 2-HP ((A-5) in Fig. 5), higher MS peaks corresponding to the cleaved ring **6** after cleavage of the NHS ester were observed in MALDI–TOF–MS spectra, and there was no series stemming from the bromide substitution (C-6).

The resultant solution was just washed with water to remove 2-HP, because the removal process was found to be necessary to accomplish the next esterification process. Subsequently, methacryloyl chloride was reacted with the hydroxy group in NHS of **6**, leading to regeneration of NHS-ester linker attaching the vinyl group. The reaction mixture was passed through a thin pad of neutral silica gel for desalting. In ^1^H NMR spectrum (B-7), the peaks from olefinic protons (*a*_*7*_) were observed with almost quantitative integration ratios to others and the spectrum of MALDI–TOF–MS also supported generation of opened ring **7**. All the 3 steps were highly efficient, and indeed the yield of opened ring **7** was >90% (based on **4**). The resultant compound **7** is a sequence-controlled trimer on the basis of carbon-based main chain, and next vinyl group that is connected at the terminal via the two cleavable-renewable bonds is ready to be ambulant for further sequence-regulated propagation via the iterative process.

## Discussion

Towards sequence-controlled vinyl oligomer (polymer), we newly designed an inimer molecule **1**, where an initiator for metal-catalyzed living radical polymerization and a conjugated vinyl group (methacrylate type) are connected via special linker consisting of two kinds of cleavable but renewable bonds, that is, NHS-ester and Py-SS. As shown by the spectra (^1^H NMR and MALDI–TOF–MS) of products (Fig. 5), all the processes including addition, cleavage and regeneration were almost quantitative. Importantly, though the addition process is basically chain-growth radical reaction, the design with the two types of special bonds specifically allow stepwise reaction as well as functionalization for resultant side chains. Thus, the linker is responsible not only for control of single-monomer radical addition via cyclization but also for iteration of the addition reaction.

The iterative process is akin to peptide synthesis in nature: ribosome or growing chain migrates to control propagation of one unit via amidation, and monomers (amino acids) are not arranged before polymerization but only one monomer gets close to growing terminal for the elongation. Side chains of the product could be constructed with the attacking molecules (amine and thiol) at the cleaving step, conferring high latitude or variety on the side chain functionalities. To heighten practical viewpoint, it would be possible to combine the resin-supported system like Merrifield methodology for peptide syntheses. Thus, sequence regulation for vinyl polymers where side chains are arranged in some intended sequence along a carbon-based main chain is within our reach.

The possibility to evolve into syntheses of sequence-controlled polymers is described in this paper, but the strategy involves some issues toward construction of longer sequence-controlled segment with variety of functional groups as well as higher efficiency in getting products. Indeed, there are structural limitations in terms of introduced functional groups: for instance, introduction of primary amine and thiol groups without protection is difficult, because they are used as the attacking sites for the cleavage reactions. Introduction of other kinds of cleavage/regeneration reactions would be necessary to provide diversity in functional groups as sequence-controlled side-chains. For improvement of the efficiency, we believe that the additional design would help the system progress further. One possible approach is attaching soluble polymer supporter in the linker between NHS-ester and Py-SS. Such design would allow not only easy purification via reprecipitation after each step but also recycle use of the polymer supported inimer molecule, which eventually could lead to automation process. Anyhow, the principle would open the door to development of highly functionalized synthetic polymers comparable to biopolymers.

## Methods

### General

Unless stated, all the solvents were purchased from Tokyo Chemical Industry Co., Ltd. (TCI) and used without further purification. *N*-hydroxymaleimide (>97.0%, Aldrich), 2-mercaptoethanol (>98.0%), tetrabromomethane (>99.0%), triphenylphosphine (>95.0%), sodium azide (>98%), 2-mercaptonicotinic acid (> 98.0%), propargyl alcohol (> 98.0%), N,N'-diisopropylcarbodiimide (DIC, >97.0%), 4-dimethylaminopyridine (DMAP, >99.0%), copper iodide (>99.5%, Aldrich), N,NDiisopropylethylamine (DiPEA, >98.0%), 2-bromoisobutyryl bromide (>98.0%), Cu wire (diameter: 0.64 mm, >99.999%, Aldrich), CuBr_2_ (>99.0%, Aldrich), 2,2'-bipyridyl (bpy, >99.0%, Aldrich), 4,4′-dimethoxy-2,2′-bypiridyl (MeO-bpy; >98.0%), butanethiol (TCI, > 97.0%), 2-hydroxypyridine (2-HP, >98.0%) and butylamine (>99.0%) were used as received. Triethylamine (> 99.0%) was dried overnight over calcium chloride and distilled from calcium hydride before use. Methacryloyl chroride (>80%) was distilled before use. Column chromatography was carried out using Wakosil C300 (Wako) as the stationary phase. Exceptionally, the final product **1** was purified with column chromatography with neutral silica gel N60 (Kanto Kagaku) because of lability of **1** under acidic condition.

^1^H and ^13^C NMR spectra were recorded on a JEOL JNM-ECA500 spectrometer, operating at 500 and 125 MHz, respectively. MALDI–TOF–MS analysis was performed on a Shimadzu AXIMA-CFR instrument equipped with 1.2 m linear flight tubes and a 337 nm nitrogen laser with matrix of dithranol.

Detailed synthetic procedures are reported in the Supplementary Methods.

### Model reaction: cleavage of NHS-ester of **1**

**1** (5 μmol) and 2-hydroxypyridine (2-HP, 5 μmol) were placed in glass tube, dissolved in THF (0.5 ml) and cooled at 0 °C. To this resultant solution was added a solution of *n*-butylamine (15 μmol) in THF (0.5 ml) and subsequently stirred for 3 h at 0 °C. After the reaction solution was evaporated under reduced pressure, the crude product was subjected to ^1^H NMR spectroscopy without any purification.

### Model reaction: cleavage of SS-pyridine of **1**

**1** (15 μmol) was placed in glass tube and dissolved in DCM (0.6 ml) containing acetic acid (1.5 μmol). To this resultant solution was added a solution of *n*-butanethiol (45 μmol) in EtOH (0.4 ml) and subsequently stirred for 24 h at room temperature. After the reaction solution was evaporated under reduced pressure, the crude product was subjected to ^1^H NMR spectroscopy without any purification.

## 

## Additional information

**How to cite this article:** Hibi, Y. *et al.* A strategy for sequence control in vinyl polymers via iterative controlled radical cyclization. *Nat. Commun.* 7:11064 doi: 10.1038/ncomms11064 (2016).

## Supplementary Material

Supplementary Information Supplementary Figures 1-10, Supplementary Table 1, Supplementary Methods and Supplementary Reference
