# Supplementary material for: A strategy for sequence control in vinyl polymers via iterative controlled radical cyclization
Source: Nat Commun. 2016 Mar 21;7:11064. doi: 10.1038/ncomms11064 (PMC4802161; doi:10.1038/ncomms11064)
Supplement: Supplementary Information — Supplementary Figures 1-10, Supplementary Table 1, Supplementary Methods and Supplementary Reference [file ncomms11064-s1.pdf]

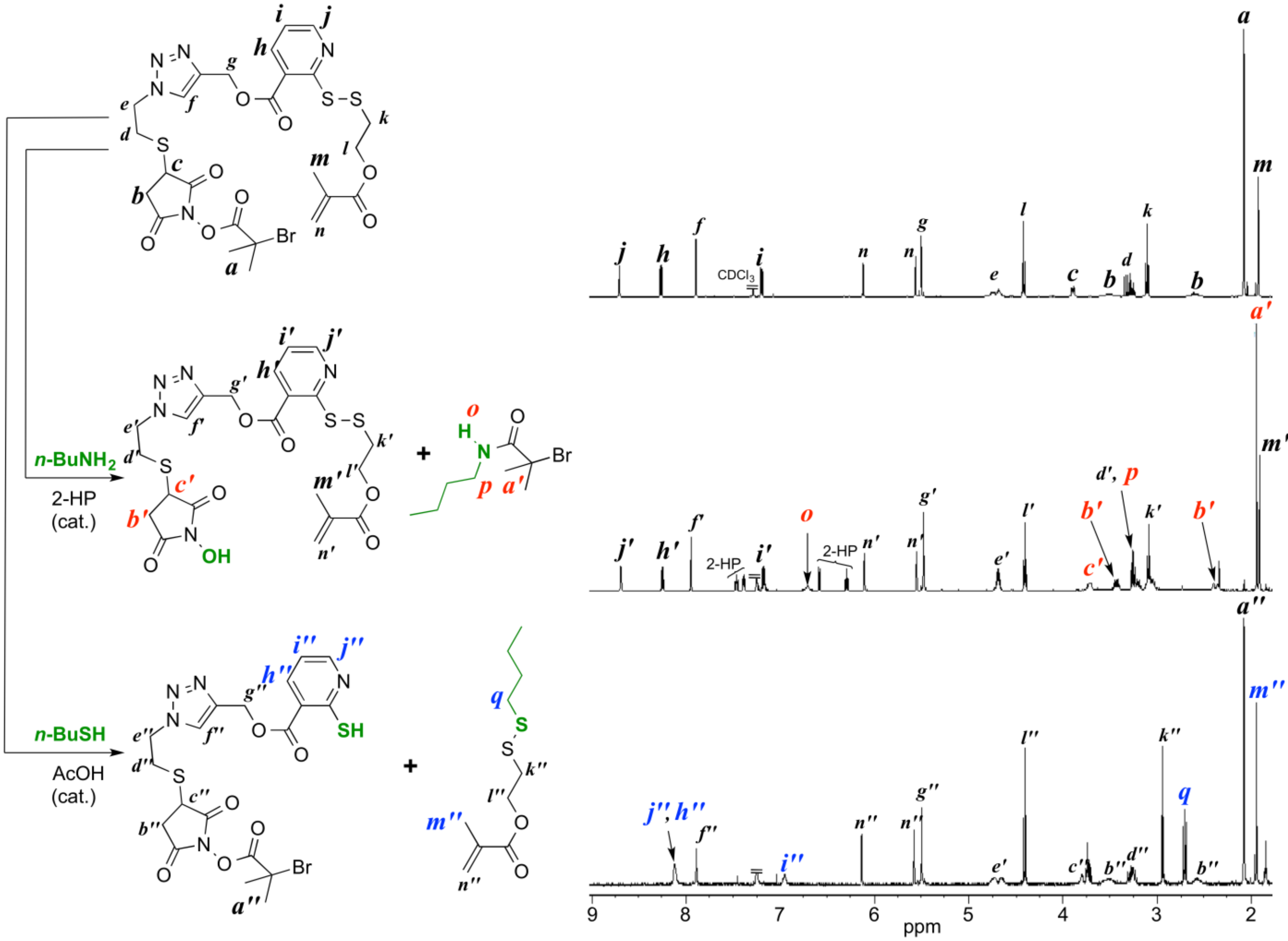

Supplementary Figure 1. Test of orthogonal cleavage of NHS-Ester and Py-SS with **1**. Cleavage of NHS-Ester: [**1**]/[*n*-BuNH$_2$]/[2-hydroxylpyridine (2-HP)] = 5/15/5 mM in DCM at 0˚C for 3 h. Cleavage of Py-SS: [**1**]$_0$/[*n*-BuSH]$_0$/[CH$_3$COOH]$_0$ = 15/45/1.5 mM in THF/EtOH (3/2 v/v%) at r.t. for 24 h. The reaction mixture was evaporated under reduced pressure, followed by direct measurement of ^{1}H NMR in CDCl$_3$ without any purification.

ESI–MS

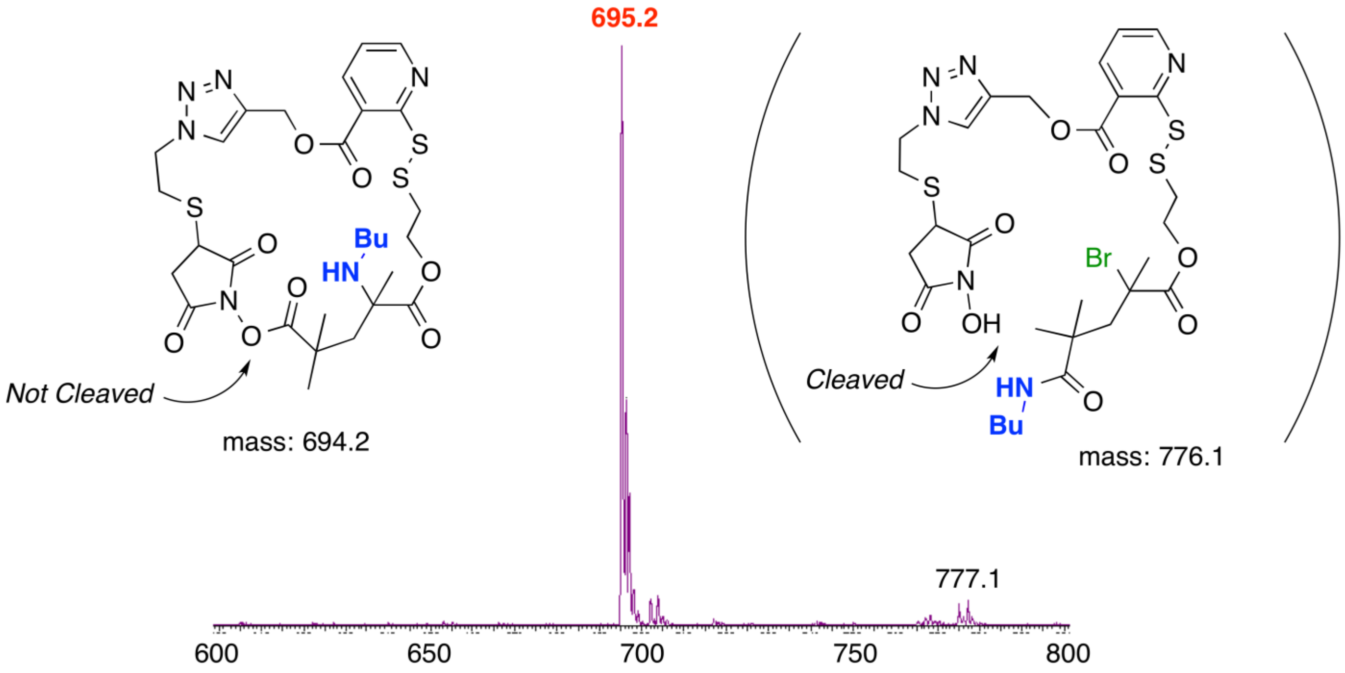

Supplementary Figure 2. ESI-MS spectrum after addition of *n*-butylamine with 2-HP into the solution of **2**: [**2**]/[*n*-butylamine]/[2-HP] =10/12/10 mM in DCM at 0 ˚C.

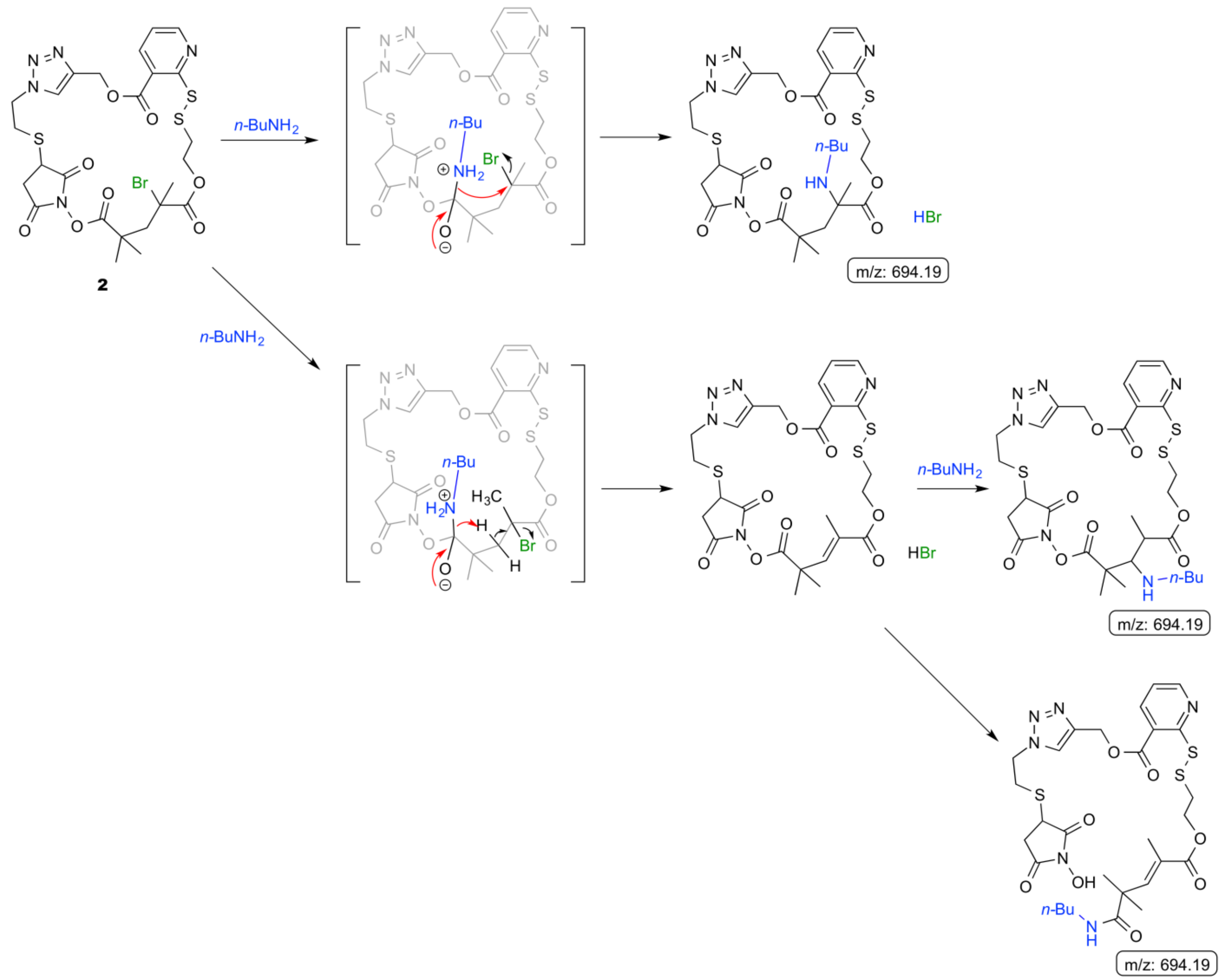

Supplementary Figure 3. Plausible reactions on the addition of *n*-BuNH$_2$ into the solution of **2**: [**2**]/[*n*-BuNH$_2$]/[2-HP] =10/12/10 mM in DCM at 0 ˚C.

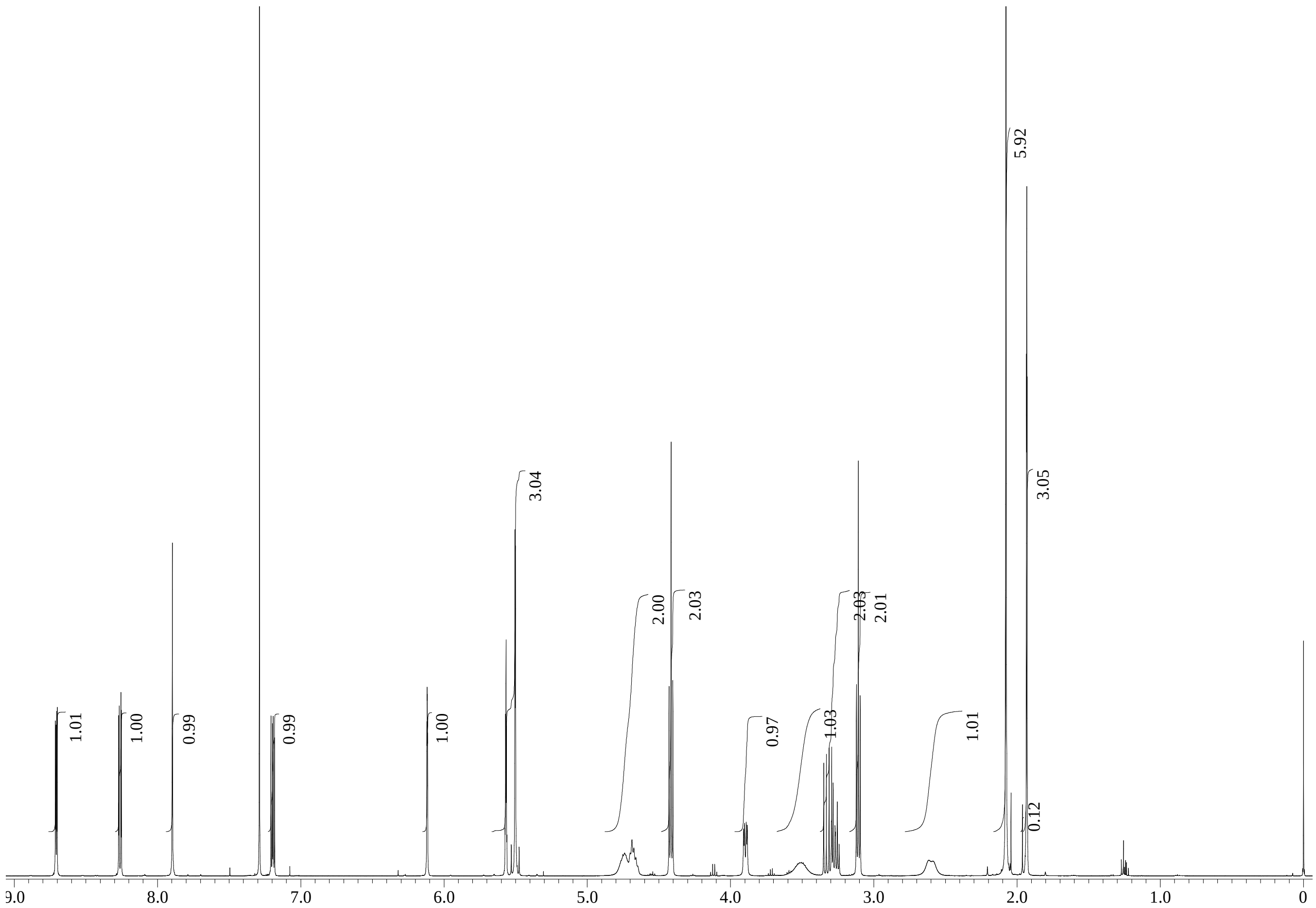

Supplementary Figure 4. ^{1}H NMR spectrum of compound **1** (500 MHz, $CDCl_3$, r.t.)

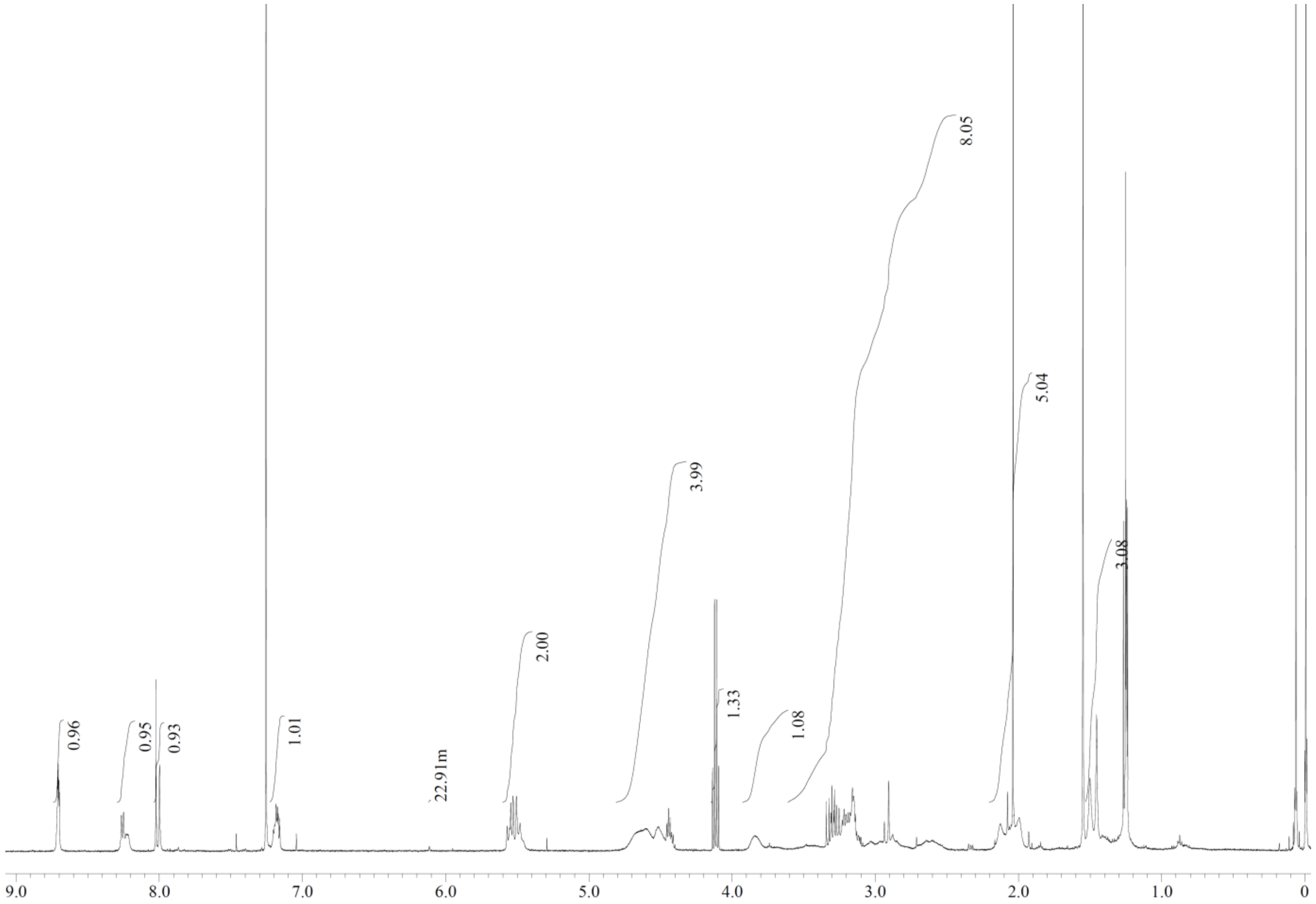

Supplementary Figure 5. ^{1}H NMR spectrum of compound **2** (500 MHz, $CDCl_3$, r.t.)

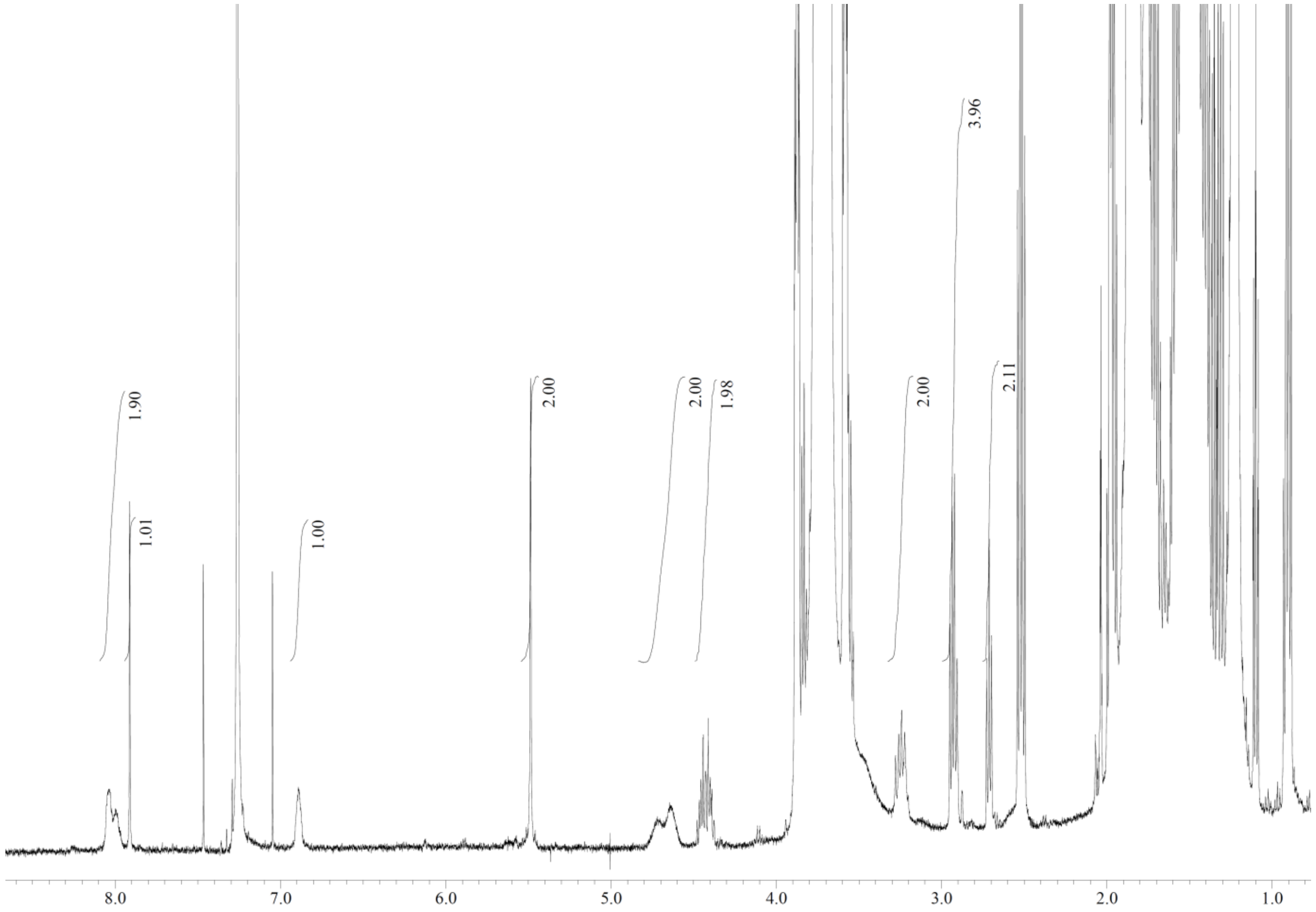

Supplementary Figure 6. ^{1}H NMR spectrum of compound **3** (500 MHz, $CDCl_3$, r.t.)

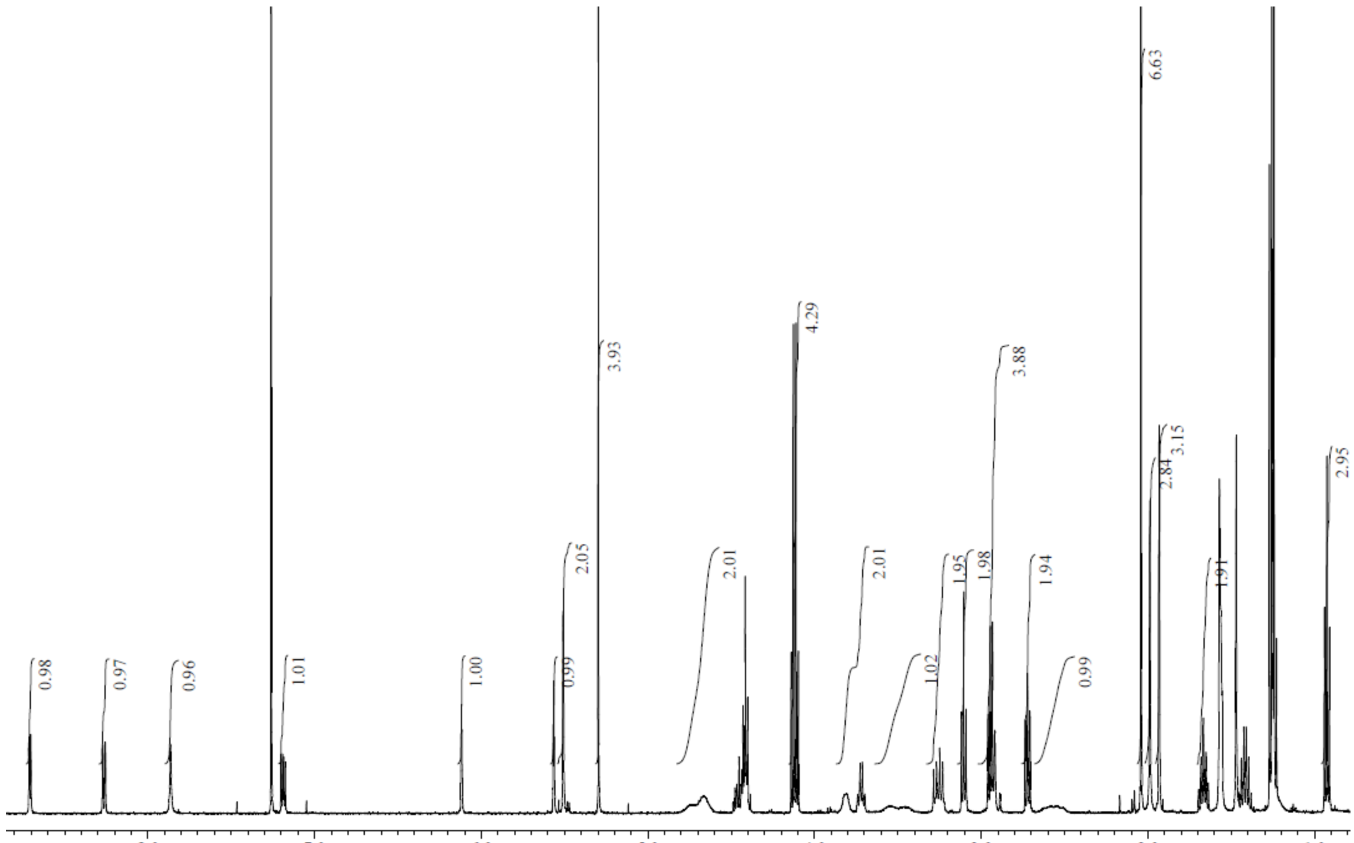

Supplementary Figure 7. ^{1}H NMR spectrum of compound **4** (500 MHz, $CDCl_3$, r.t.)

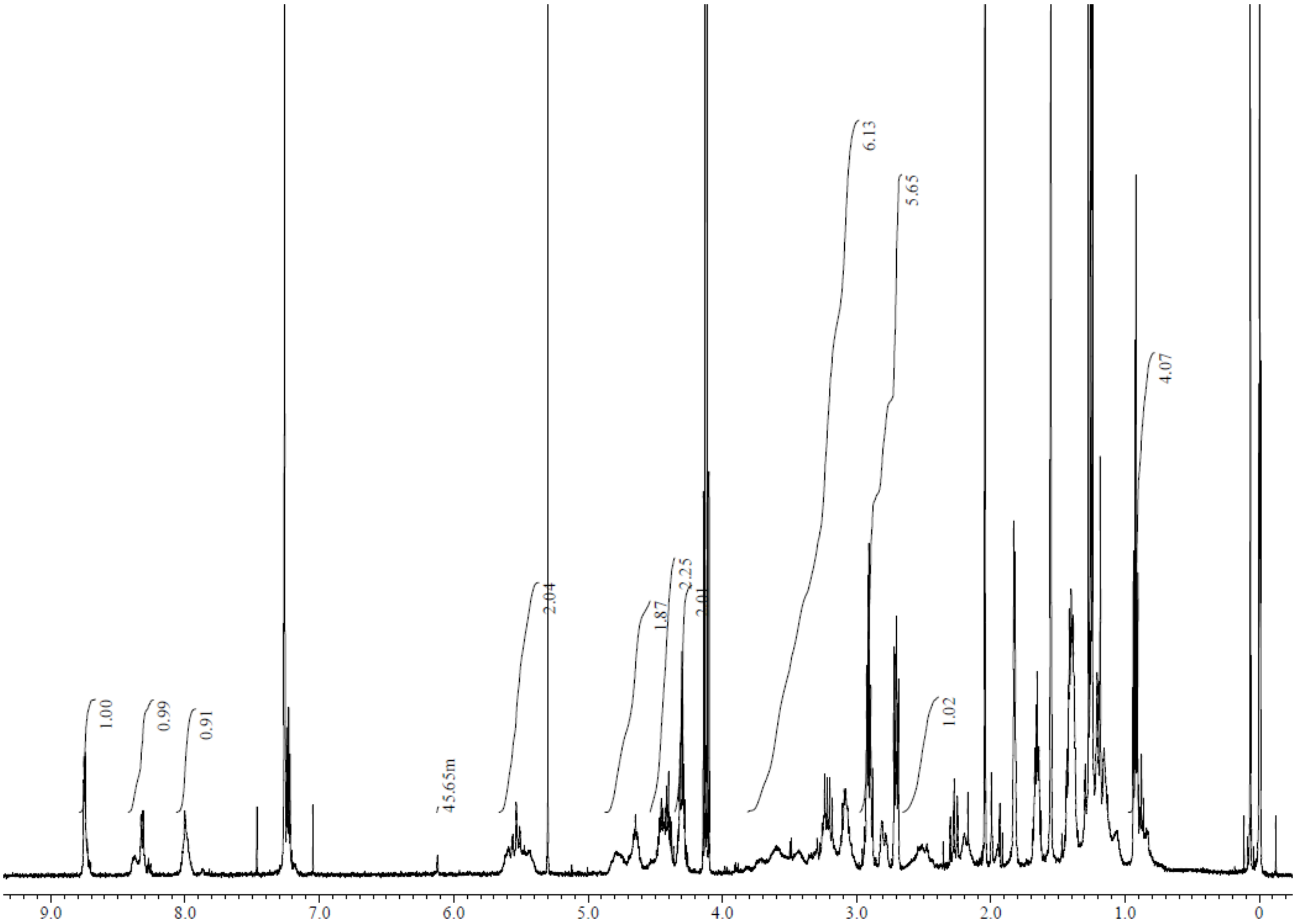

Supplementary Figure 8. ^{1}H NMR spectrum of compound **5** (500 MHz, $CDCl_3$, r.t.)

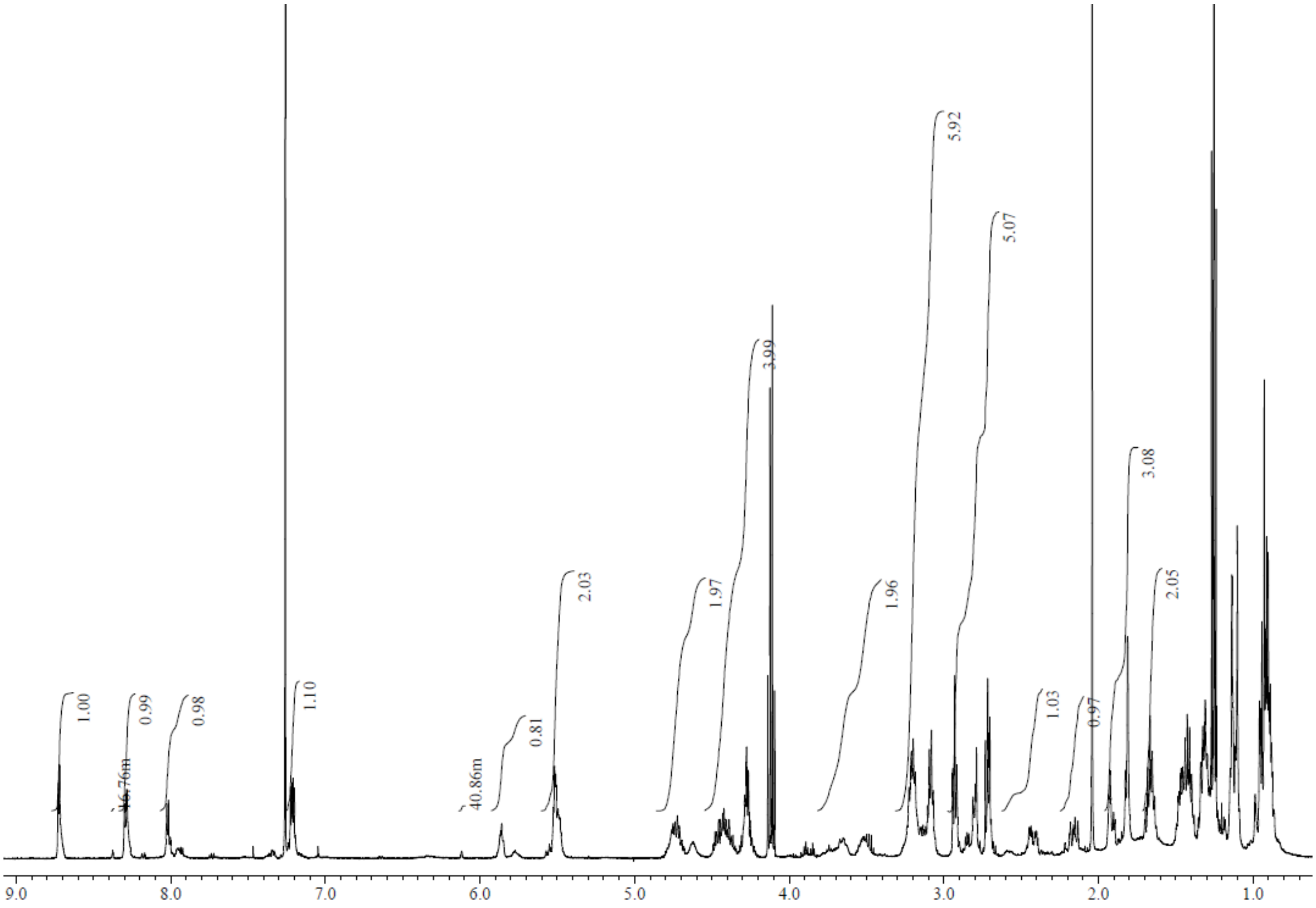

Supplementary Figure 9. ^{1}H NMR spectrum of compound **6** (500 MHz, $CDCl_3$, r.t.)

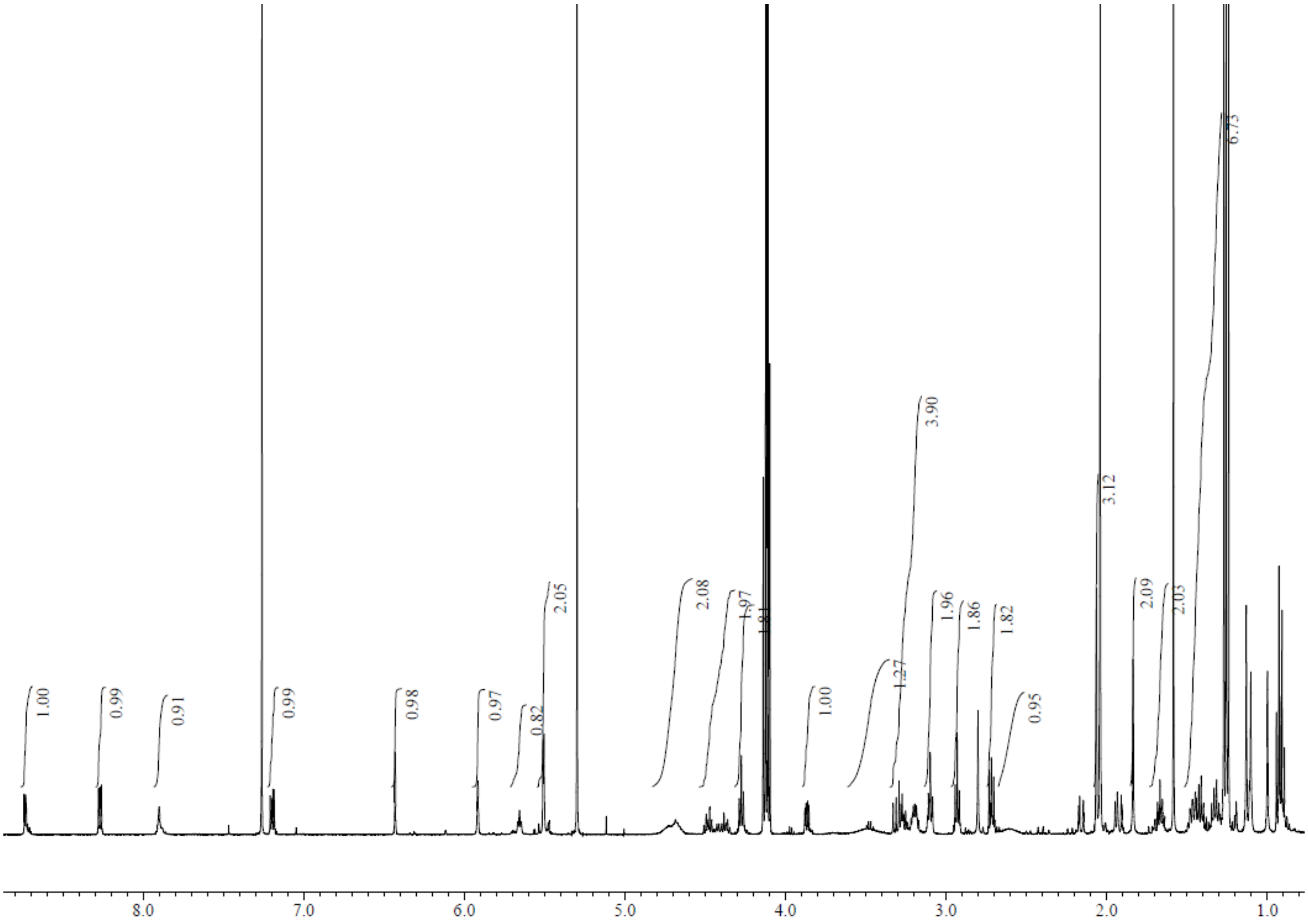

Supplementary Figure 10. ^{1}H NMR spectrum of compound **7** (500 MHz, $CDCl_3$, r.t.)

Supplementary Table 1. Screening of Condition for Metal-Catalyzed Radical Cyclization of **1**[a]

Entry	Catalyst	Solvent	$[Cat.]_0/[\mathbf{1}]_0$ mM	Conv., % (CH_2=C) [b]	Speculated Side Reactions [c]
1	$Ru(Cp^*)Cl(PPh_3)_2$	Toluene	1/10	0	Cleavage of S–S
2	$Ru(Ind)Cl(PPh_3)_2$	Toluene	1/10	0	Cleavage of S–S
3	$CuBr/CuBr_2$/bpy[d]	Anisole	2.5/10	39	No Side Reaction
4	$CuBr/CuBr_2$/bpy[d]	DMF	2.5/10	60	Decomposition[e]
5	$CuBr/CuBr_2$/bpy[d]	DMSO	2.5/10	60	Decomposition[e]
6	$CuBr/CuBr_2$/bpy[d]	Cy[f]	2.5/10	57	Dispro.[g] Dimerization
7	$CuBr/CuBr_2$/MeO-bpy[d]	Cy[f]	2.5/10	75	Dispro.[g] Dimerization
8	$Cu/CuBr_2$/MeO-bpy[h]	Cy[f]	1/10	92	Dispro.[g] Dimerization
9	$Cu/CuBr_2$/MeO-bpy[h]	Cy[f]/Toluene[i]	1/10	100	Dimerization
10	$Cu/CuBr_2$/MeO-bpy[h]	Cy[f]/Toluene[i]	0.18/2.5	100	No Side Reaction

[a] The reactions were performed at 60˚C for 18 h. [b] Consumption of the vinyl protons was determined from integration ratio in ^{1}H NMR. [c] Unfavorable side reactions were speculated from spectra of ^{1}H NMR and MALDI-TOF-MS. [d] $CuBr/CuBr_2$/ligand = 2.0/0.5/5.0 mM. [e] No peaks were observed around m/z = 704 in MALDI-TOF-MS. [f] Cy: Cyclohexanone. [g] Dispro.: disproportionation [h] $CuBr_2$/ligand = 1.0/2.0 mM with Cu wire (1 mm width). [i] Cyclohexanone/Toluene = 2/3 v/v%.

Supplementary Methods

Synthesis of Inimer (1)

Synthesis of 1-hydroxy-3-((2-hydroxyethyl)thio)pyrrolidine-2,5-dione (A)

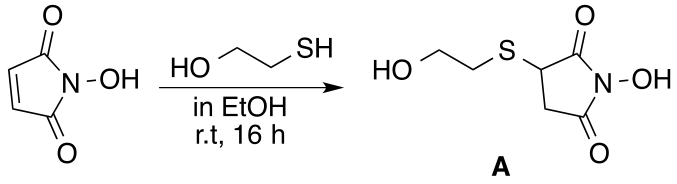

N-hydroxymaleimide (2.16 g, 19.1 mmol) was placed in round-bottom flask under nitrogen and then dissolved in EtOH (10 mL). To the resultant solution, 2-mercaptoethanol was added and subsequently stirred for 16 h at r.t. After the solvent was removed under reduced pressure, the crude product was purified with silica column chromatography (DCM:MeOH = 10:1 as the eluent) to yield 1-hydroxy-3-((2-hydroxyethyl)thio)pyrrolidine-2,5-dione as slightly yellow oil (**A**: 3.33 g, 17.4 mmol, 96% yield). ^{1}H NMR (in *d*-methanol): 3.97 (dd, 1H), 3.77 (m, 2H), 3.18 (q, 1H), 3.05 (q, 1H), 2.85 (q, 1H), 2.52 (dd, 1H).

Synthesis of 3-((2-bromoethyl)thio)-1-hydroxypyrrolidine-2,5-dione (B)

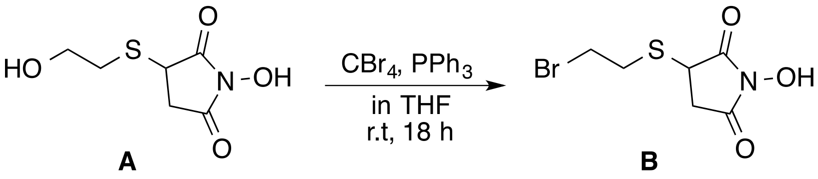

The compound **A** (3.33g, 17.4 mmol) and tetrabromomethane (6.92 g, 20.9 mmol) were placed in round-bottom flask under argon and dissolved in THF (20 mL). To the resultant solution, a solution

of triphenylphosphine (5.48 g, 20.9 mmol) in THF (20 mL) was added at 0˚C under argon, and subsequently stirred for 18 h at room temperature. After the reaction was quenched with MeOH, the reaction mixture was concentrated under reduced pressure. The obtained crude product was purified with silica column chromatography (DCM:MeOH = 100:5 as eluent) but the by-product of triphenylphosphine oxide cannot be separated from the objective product. The obtained product [3-((2-bromoethyl)thio)-1-hydroxypyrrolidine-2,5-dione (**B**), 14.3 mmol, 82% yield] was used for next reaction without further purifications. ^{1}H NMR (in $CDCl_3$): 3.63 (m, 2H), 3.53 (m, 1H), 3.40 (m, 1H), 3.12 (m, 1H), 3.02 (q, 1H), 2.38 (dd, 1H).

Synthesis of 3-((2-azidoethyl)thio)-1-hydroxypyrrolidine-2,5-dione (C)

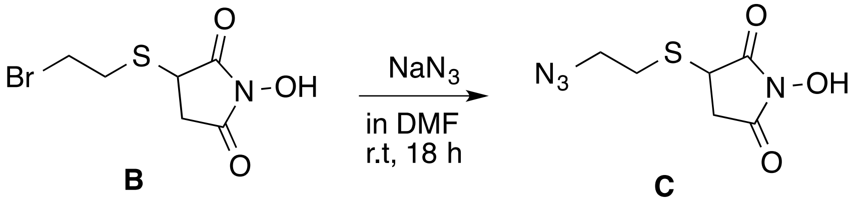

The compound **B** (3.56g, 14.0 mmol) was placed in round-bottom flask and dissolved in DMF (40 mL). Before addition of NaN_3, the DMF solution was placed under reduced pressure for 30 min to completely remove residual DCM in the product of **B**, followed by refill with argon. Sodium azide (1.21 g, 15.5 mmol) was placed in another round-bottom flask under argon. To the flask of sodium azide, the DMF solution of **B** was transferred and subsequently stirred for 18 h at room temperature. After the reaction mixture was concentrated under reduced pressure at 40˚C, the reaction was quenched with water. The aqueous layer was extracted 4 times with ethyl acetate, and then the organic phase was washed with brine and dried over Na_2SO_4. After concentration under reduced pressure, the crude product was purified with silica column chromatography (DCM:MeOH = 100:5) to give 3-((2-

azidoethyl)thio)-1-hydroxypyrrolidine-2,5-dione (**C**: 8.4 mmol, 60%). ^{1}H NMR (in $CDCl_3$): 3.83 (m, 1H), 3.61 (m, 2H), 3.17 (m, 2H), 2.93 (m, 1H), 2.49 (dd, 1H).

Synthesis of 2-((methoxycarbonyl)disulfanyl)ethyl methacrylate (D)[1)]

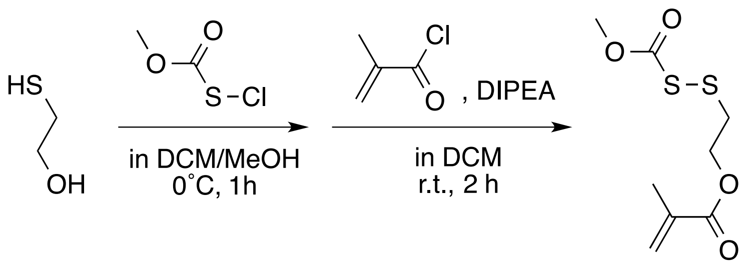

D

To a solution of methoxycarbonylsulfenyl choride (1.42 mL, 15.6 mmol) in MeOH (30 mL) was added a solution of 2-mercaptoethanol (15.6 mmol in DCM; 15 mL) at 0˚C under dry argon, and the solution was subsequently stirred for 1 h. Then, the solvents were removed by evaporation, and to completely remove methanol, 20 mL of dehydrated toluene was added and evaporated again. This intermediate product was dissolved in DCM (20 mL) and treated with methacryloyl chloride (1.90 mL, 20 mmol) for 2 hours at r.t. in the presence of DIPEA (3.48 mL, 20 mmol). After the reaction was quenched by adding MeOH, the solvents were removed under reduced pressure. The resultant crude product was purified by silica column chromatography (ethyl acetate:hexane = 1:4 as a eluent), to yield a colorless oil [2-((methoxycarbonyl)disulfanyl)ethyl methacrylate, **D**: 3.3 g, 14.1 mmol, yield 91%]. ^{1}H NMR ($CDCl_3$): □ 6.14 (s, 1H), 5.64 (s, 1H), 4.39 (t 2H), 3.89 (s, 3H) 3.06(t, 2H), 1.94 (s, 3H).

Synthesis of 2-((2-(methacryloyloxy)ethyl)disulfanyl)nicotinic acid (E)

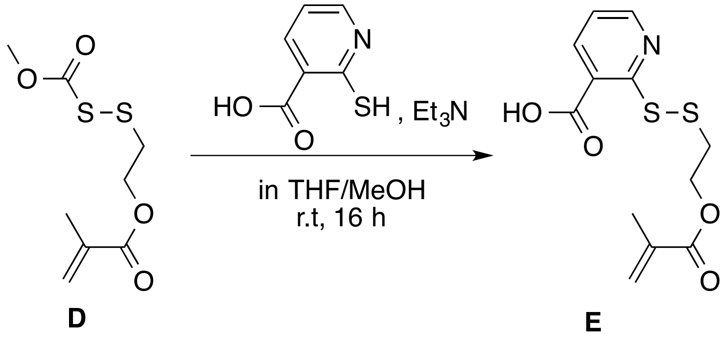

2-Mercaptonicotinic acid (0.69 g, 4.4 mmol) was placed in round-bottom flask and suspended in MeOH (4 mL) and THF (4 mL). To the resultant suspension, the compound **D** (4.0 mmol in DCM 7.3 mL) triethylamine (0.62 mL, 4.4mmol) were added at 0˚C and subsequently stirred for 16 h to give clear solution. The reaction was quenched with HCl aq. (1M, 10mL) and then the aqueous layer was extracted with the organic solvents (DCM:MeOH = 5:1) three times. The organic phase was washed with brine, dried over Na_2SO4, and then evaporated to give 2-((2-(methacryloyloxy)ethyl)disulfanyl)nicotinic acid (**E**, 1.18g, 3.90 mmol, 95% yield). ^{1}H NMR (in $CDCl_3$): 8.69 (d, 1H), 8.30 (d, 1H), 7.23 (q, 1H), 6.12 (s, 1H), 5.58 (s, 1H), 4.40 (t, 2H), 3.10 (t, 2H), 1.93 (s, 3H).

Synthesis of prop-2-yn-1-yl 2-((2-(methacryloyloxy)ethyl)disulfanyl)nicotinate (F)

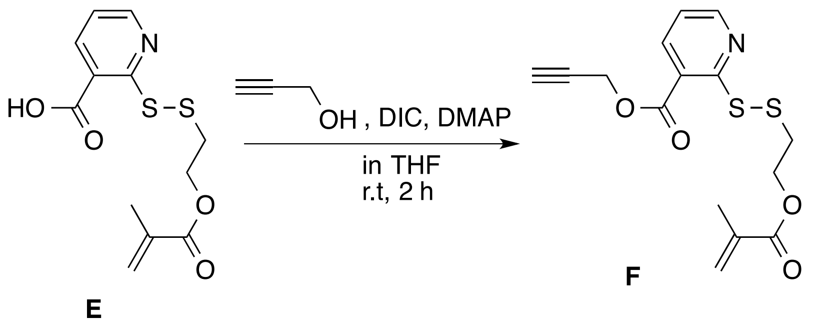

The compound **E** (1.5 g, 4.7 mmol) and 4-dimethylaminopyridine (DMAP; 57 mg, 0.47 mmol) were placed in round-bottom flask under argon and dissolved in THF (15 mL). To the resultant solution, propargyl alcohol (0.34 mL, 5.7 mmol) and *N,N'*-diisopropylcarbodiimide (DIC; 0.89 mL, 5.7 mmol) were added at 0˚C and subsequently stirred at 0˚C for 2 h. Then the reaction mixture was evaporated under reduced pressure to give crude product which was purified with silica column chromatography (DCM:MeOH = 100:5 as the eluent) to yield prop-2-yn-1-yl 2-((2-(methacryloyloxy)ethyl)disulfanyl)nicotinate (**F**, 1.4 g, 4.3 mmol, 92 %). ^{1}H NMR (in $CDCl_3$): 8.72 (d, 1H), 8.29 (d, 1H), 7.21 (q, 1H), 6.12 (s, 1H), 5.56 (s, 1H), 4.95 (s, 2H), 4.41 (t, 2H), 3.11 (t, 2H), 2.55 (t, 1H), 1.93 (s, 3H).

Synthesis of (1-(2-((1-hydroxy-2,5-dioxopyrrolidin-3-yl)thio)ethyl)-1H-1,2,3-triazol-4-yl)methyl 2-((2-(methacryloyloxy)ethyl)disulfanyl)nicotinate (G)

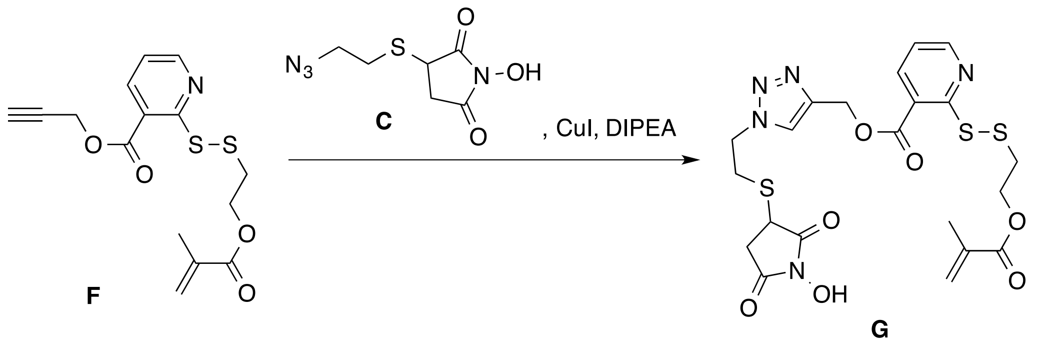

CuI (30 mg, 0.17 mmol) was placed in round-bottom flask under argon. To the flask of CuI, a THF solution of **C** (1.65 mmol) and **F** (1.98 mmol) was added followed by addition of *N,N*-Diisopropylethylamine (DiPEA; 6.6 mL, 0.66 mmol). The resultant reaction mixture was stirred for 24 h, and then the reaction was quenched with NH_4Cl aq. The aqueous phase was extracted with ethyl acetate 3 times. Then, the organic layers were combined, washed with brine and dried over Na_2SO_4. After evaporation of solvents, the crude product was purified with silica column chromatography (DCM:MeOH = 100:8 as the eluent) to give (1-(2-((1-hydroxy-2,5-dioxopyrrolidin-3-yl)thio)ethyl)-1H-1,2,3-triazol-4- yl)methyl 2-((2-(methacryloyloxy)ethyl)disulfanyl)nicotinate (**G**, 0.8 g, 1.44 mmol, 88% yield). ^{1}H NMR (in $CDCl_3$): 8.70 (d, 1H), 8.27 (d, 1H), 7.88 (s, 1H), 7.18 (q, 1H), 6.12 (s, 1H), 5.56 (s,

1H), 5.47 (s, 2H), 4.69 (m, 1H), 4.65 (m, 1H), 4.41 (t, 2H), 3.54 (dd, 1H), 3.45 (m, 1H), 3.19 (m, 1H), 3.10 (t, 2H), 2.98 (m, 1H), 2.33 (dd, 1H), 1.93 (s, 3H).

Synthesis of (1-(2-((1-((2-bromo-2-methylpropanoyl)oxy)-2,5-dioxopyrrolidin-3-yl)thio)ethyl)-1H-1,2,3-triazol-4-yl)methyl 2-((2-(methacryloyloxy)ethyl)disulfanyl) nicotinate (1)

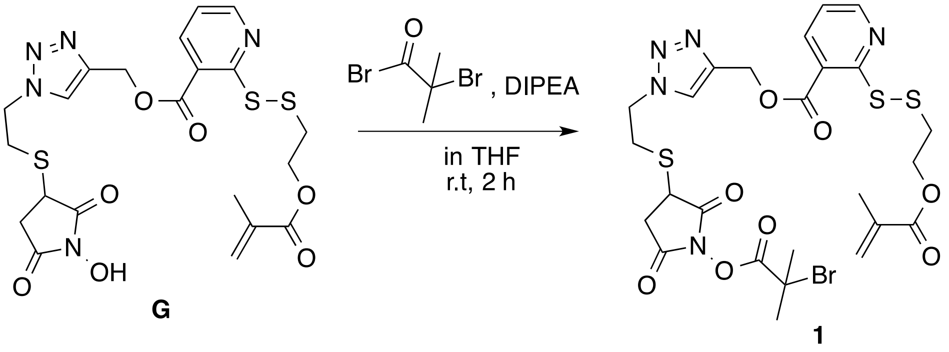

The compound **G** (0.874 mmol) was placed in round-bottom flask and dissolved in THF (5 mL). To the resultant solution, DiPEA (0.222 mL, 1.31 mmol) and 2-bromoisobutyryl bromide were added at 0˚C and subsequently stirred at rt for 2 h. Then, the reaction mixture was quenched with methanol, washed with water (extraction: 4 tims with ethyl acetate) and then dried over Na_2SO_4. After removal of solvents under reduced pressure, the crude product was purified with neutral silica column chromatography (DCM:ethyl acetate = 3:1 as the eluent) to give (1-(2-((1-((2-bromo-2-methylpropanoyl)oxy)-2,5-dioxopyrrolidin-3-yl)thio)ethyl)-1H-1,2,3-triazol-4-yl)methyl 2-((2-(methacryloyloxy)ethyl)disulfanyl) nicotinate (**1**, 0.53 g, 0.76 mmol, 87%). ^{1}H NMR (in $CDCl_3$): 8.71 (d, 1H), 8.25 (d, 1H), 7.90 (s, 1H), 7.19 (q, 1H), 6.12 (s, 1H), 5.57 (s, 1H), 5.51 (d, 2H), 4.7 (br m, 2H), 4.41 (t, 2H), 3.87 (dd, 1H), 3.52 (br s, 1H), 3.30 (m, 2H), 3.10 (t, 2H), 2.59 (br s, 1H), 2.08 (s, 6H), 1.93 (s, 3H): MALDI-TOF-MS analysis: 703.1 m/z (calculated mass: 703.1). The ^{1}H NMR spectrum of compound **1** is shown in Supplementary Figure 4.

1st Radical Addition (Cyclization) with 1: Synthesis of 2

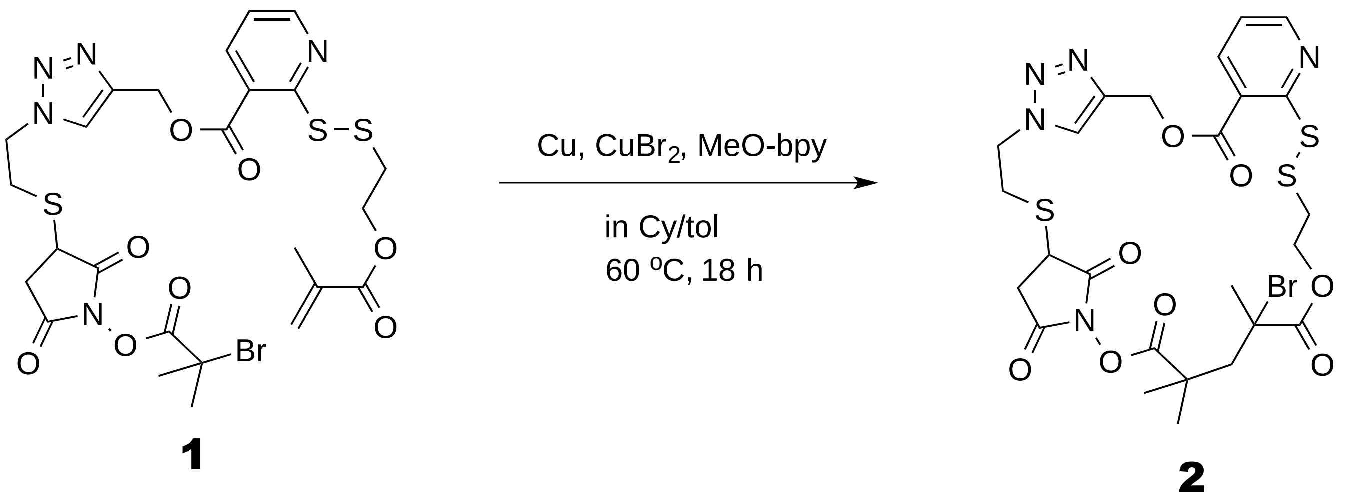

$CuBr_2$ (1.4 mg, 6.3 μmol) and 4,4'-dimethoxy-2,2'-bypiridyl (MeO-bpy; 2.7 mg, 12.5 μmol) were placed in round-bottom flask under argon and dissolved in cyclohexanone (Cy; 10 mM) to prepare stock solution of catalyst. Cu wire (d: 0.66 mm, l: 1 mm) was placed in another round-bottom flask under argon. To this flask of Cu wire, toluene (5.5 mL), cyclohexanone (1.1 mL), a stock solution of **1** (25 μmol in tol 0.5 mL) in toluene and a stock solution of catalyst (2.88 mL) prepared as above were added in this order. The resultant mixture was heated at 60 ˚C and stirred for 18 h. After concentration under reduced pressure, the residual olefinic protons (6.12 ppm) in ^{1}H NMR spectroscopy (in $CDCl_3$) was not detected, indicating the reaction proceeded quantitatively. Passing the crude through a thin pad of neutral silica gel eluted with DCM:EtOAc = 3:1 yielded **2** as colorless oil (17 mg, 24 μmol). The ^{1}H NMR spectrum of compound **2** is shown in Supplementary Figure 5.

Selective Cleavage of PySS in 2: Synthesis of 3

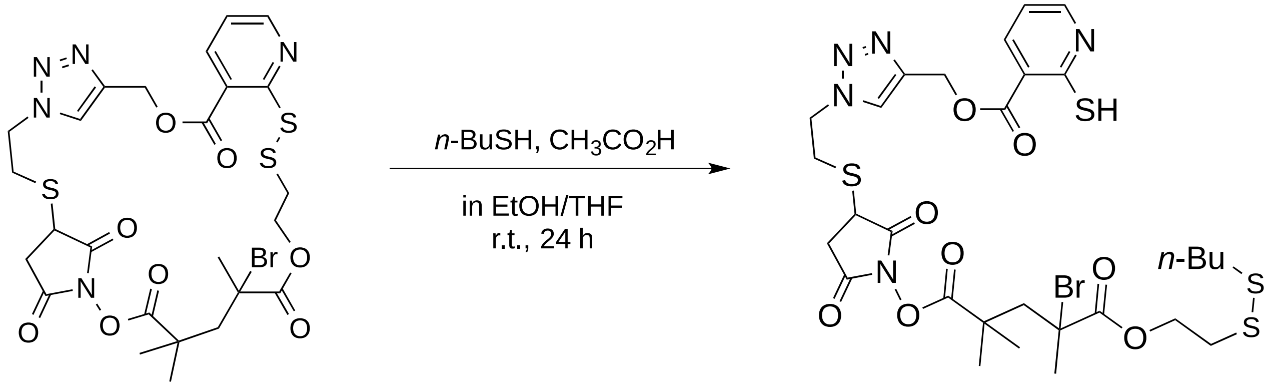

2 **3**

2 (17 mg, 24 μmol) was dissolved in DCM (0.6 mL) containing acetic acid (1.5 μmol). To this resultant solution was added a solution of *n*-butanethiol (75 μmol) in EtOH (0.4 mL) and subsequently stirred for 24 hours at r.t. The quantitative conversion of **2** to **3** was confirmed by clear peak shift of pyridyl protons in ^{1}NMR spectrum. Evaporation of resulting reaction solution afforded crude product of **3** as yellow oil, which is used in the next reaction without further purification. The ^{1}H NMR spectrum of crude compound **3** is shown Supplementary Figure 6.

Regeneration of PySS for 3: Synthesis of 4

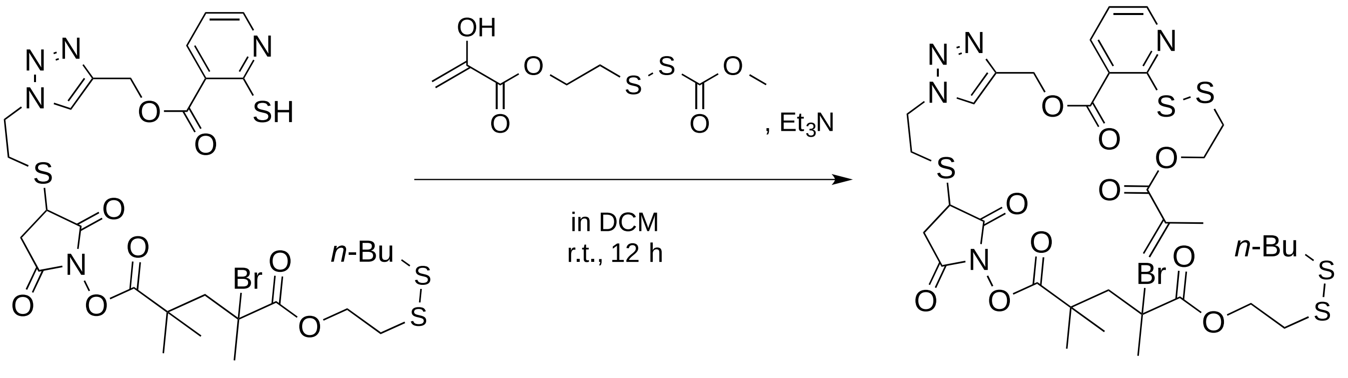

3 **4**

The obtained **3** was dissolved in THF 0.8 mL and EtOH 0.4 mL. To this resulting solution, 2-((methoxycarbonyl) disulfanyl)ethyl methacrylate (25 μmol) in DCM (0.1 mL) and triethyl amine (5 μmol) in EtOH (0.1 mL) were added in this order. After reaction for 12 h, the color of yellow from 2-pyridyl thiol moiety completely vanished and the reaction conversion reached to 100%, confirmed with ^{1}H NMR spectroscopy. After concentration under reduced pressure, the crude product was purified by neutral silica column chromatography eluted with DCM/AcOEt = 6/1. **4** was obtained as colorless oil (21mg; 23 μmol, 92% yield based on **1** through 3 steps). The ^{1}H NMR spectrum of compound **4** is shown in Supplementary Figure 7**.**

2nd Radical Addition (Cyclization) with 4: Synthesis of 5

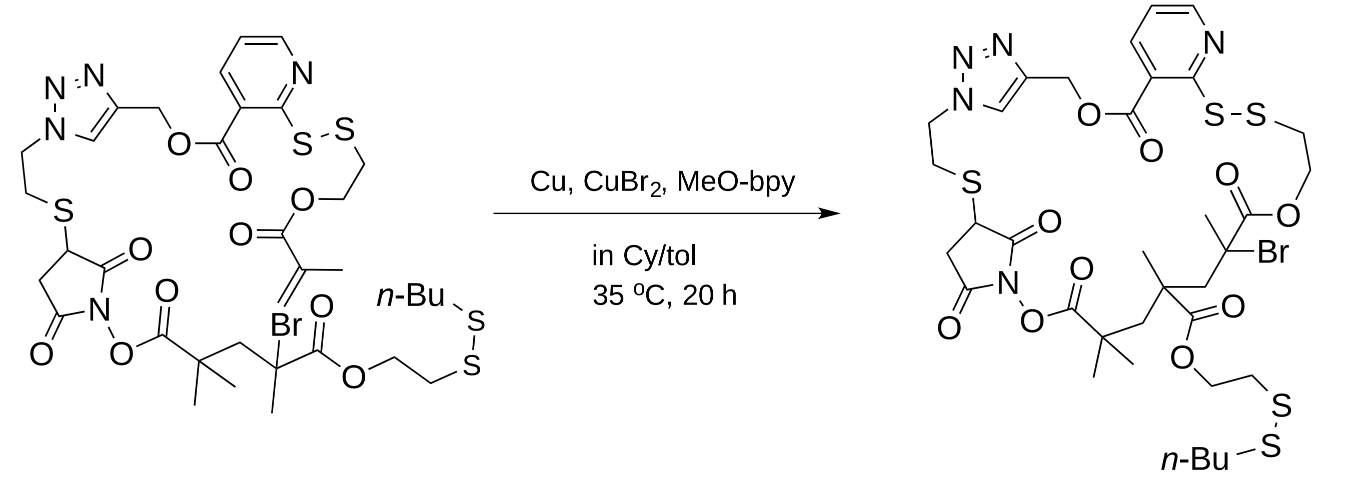

4 **5**

$CuBr_2$ (2.5 mg, 11 μmol) and 4,4'-dimethoxy-2,2'-bypiridyl (MeO-bpy; 4.9 mg, 22 μmol) were placed in round-bottom flask under argon and dissolved in cyclohexanone (Cy; 30 mM) to prepare stock solution of catalyst. To another flask of **4** (75 mmol), toluene (20 mL), Cu wire (d: 0.66 mm, l: 1 mm) and the stock solution of catalyst (10 mL) prepared as above were added in this order. The resultant mixture was heated at 35 ˚C and stirred for 24 h. After concentration under reduced pressure, the reaction conversion was calculated from peak area ratio between residual olefinic protons (6.12 ppm) and pyridyl proton (8.71 ppm) unrelated to the reaction with 1H NMR spectroscopy (in CDCl3), confirming that the reaction conversion reached to 95%. Passing the crude product through a thin pad of neutral silica gel eluted with DCM:EtOAc = 5:1 yielded **5** as colorless oil (66mg; 70 μmol, 93% yield). The ^1H NMR spectrum of compound **5** is shown in Supplementary Figure 8.

Selective Cleavage of NHS-Ester in 5: Synthesis of 6

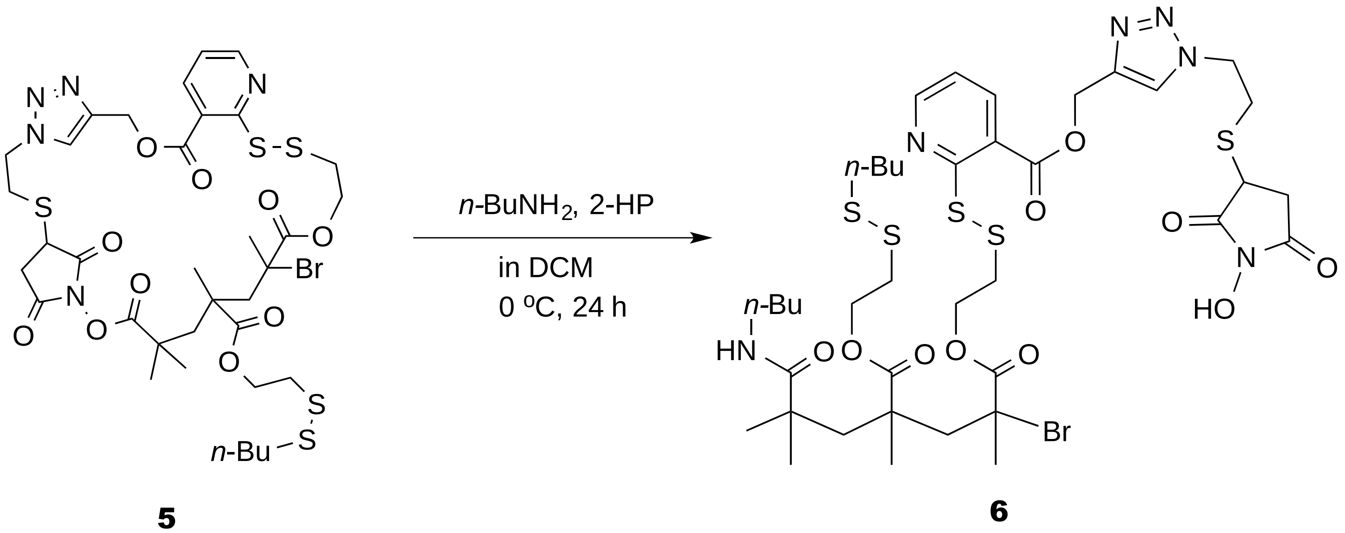

5 (70 μmol) and 2-hydroxypyridine (2-HP, 70 μmol) were placed in glass tube, dissolved in THF (3 mL) and cooled at 0 ˚C. To this resultant solution was added a solution of *n*-butylamine (210 μmol) and subsequently stirred for 24 h at 0 ˚C. The reaction solution was washed with water and extracted with EtOAc 3 times. The organic solution was dried over Na_2SO4 and then concentrated under reduced pressure to afford **6** (68 mg; 67 μmol, 95% yield). Without further purification, the crude compound **6** was used in the next step. The ^{1}H NMR spectrum of compound **6** is shown in Supplementary Figure 9.

Regeneration of NHS-Ester for 6: Synthesis of 7

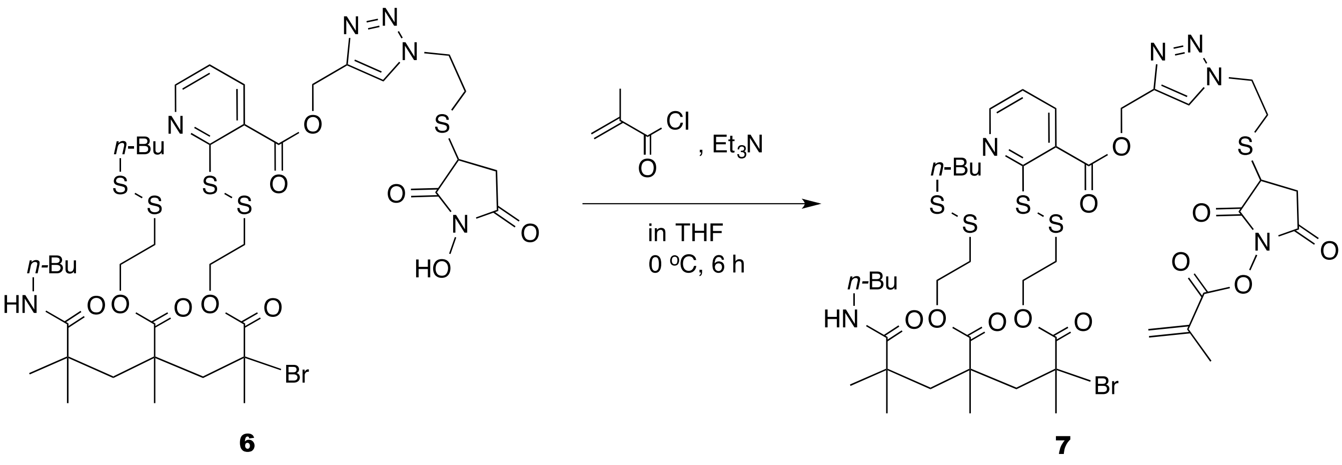

To the flask of **6** (64 μmol), THF (1.0 mL), triethylamine (210 μmol) and methacryloyl chloride (210 μmol) were added in this order at 0 °C. After stirring the mixture for 6 h, the reaction was quenched by adding small amount of methanol. The reaction mixture was directly passed through a thin pad of silica-gel for desalting. After the solvents were removed under reduced pressure, **7** was obtained as colorless oil (66 mg; 61 mmol; 91%). The ^{1}H NMR spectrum of compound **7** is in Supplementary Figure 10.

Supplementary Reference

1) S. J. Brois, J. F. Pilot and H. W. Barnum, *J. Am. Chem. Soc.* **1970**, *92(26)*, 7629-76331.
